# Enhancer variants reveal a conserved transcription factor network governed by PU.1 during osteoclast differentiation

**DOI:** 10.1038/s41413-018-0011-1

**Published:** 2018-03-28

**Authors:** Heather A. Carey, Blake E. Hildreth, Jennifer A. Geisler, Mara C. Nickel, Jennifer Cabrera, Sankha Ghosh, Yue Jiang, Jing Yan, James Lee, Sandeep Makam, Nicholas A. Young, Giancarlo R. Valiente, Wael N. Jarjour, Kun Huang, Thomas J. Rosol, Ramiro E. Toribio, Julia F. Charles, Michael C. Ostrowski, Sudarshana M. Sharma

**Affiliations:** 1 Department of Cancer Biology and Genetics and Comprehensive Cancer Center, The Ohio State University Wexner Medical Center, Columbus, OH 43210 USA; 2 College of Veterinary Medicine, The Ohio State University, Columbus, OH 43210 USA; 3 Department of Biochemistry and Molecular Biology and Hollings Cancer Center, Medical University of South Carolina, Charleston, SC 29425 USA; 4 Division of Rheumatology, Immunology and Allergy, Department of Medicine, Brigham and Women’s Hospital and Harvard Medical School, Boston, MA USA; 5 Division of Rheumatology and Immunology, Department of Internal Medicine, The Ohio State University Wexner Medical Center, Columbus, OH 43210 USA; 6 Department of Biomedical Informatics, The Ohio State University Wexner Medical Center, Columbus, OH 43210 USA

## Abstract

Genome-wide association studies (GWASs) have been instrumental in understanding complex phenotypic traits. However, they have rarely been used to understand lineage-specific pathways and functions that contribute to the trait. In this study, by integrating lineage-specific enhancers from mesenchymal and myeloid compartments with bone mineral density loci, we were able to segregate osteoblast- and osteoclast (OC)-specific functions. Specifically, in OCs, a PU.1-dependent transcription factor (TF) network was revealed. Deletion of PU.1 in OCs in mice resulted in severe osteopetrosis. Functional genomic analysis indicated PU.1 and MITF orchestrated a TF network essential for OC differentiation. Several of these TFs were regulated by cooperative binding of PU.1 with BRD4 to form superenhancers. Further, PU.1 is essential for conformational changes in the superenhancer region of Nfatc1. In summary, our study demonstrates that combining GWASs with genome-wide binding studies and model organisms could decipher lineage-specific pathways contributing to complex disease states.

## Genetics: Cellular fate dictates osteoporosis risk

Genetic variation in non-coding regions of DNA could raise osteoporosis risk by affecting osteoclast differentiation. Osteoporosis occurs when the normal process of bone remodeling by osteoblasts and osteoclasts falls out of balance. Genome-wide association studies (GWAS) have identified numerous single nucleotide polymorphisms (SNPs) associated with osteoporosis, but how these affect specific cell types was unclear. Sudarshana Sharma and Michael Ostrowski at the Medical University of South Carolina and colleagues wondered if variations in non-coding ‘enhancer’ regions of DNA, might shed light on the molecular underpinnings of osteoporosis. So, they overlaid SNPs associated with reduced bone mineral density onto enhancers in mesenchymal and myeloid cells—the precursors of osteoblasts and osteoclasts—identifying a transcription factor network in myeloid cells that drives the differentiation of osteoclasts. When this was disrupted in mice, severe defects in osteoclast differentiation and function resulted.

## Introduction

The availability of datasets from large genome-wide association studies (GWAS) has contributed immensely to our understanding of the effect of human genetic variations on the pathophysiology of complex traits. Over 90% of phenotypically associated single nucleotide polymorphisms (SNPs) reported in the GWAS catalog^1^ are present in non-coding regions within the genome.^2^ Advances in the field of genomics has enabled us to better understand the functional significance of SNPs by integrating gene expression, GWAS, and functional genomics.^3,4^ Genomics has contributed significantly to our understanding of cellular differentiation, development, and disease. However, using GWAS as an investigative tool to analyze complex traits by pairing it with large-scale genomics and animal modeling has been rarely performed.

Osteoporosis is a multi-factorial disease that is well-studied using GWAS. Although historically associated with age-related hormonal deficiencies, other diseases, therapeutic agents, and lifestyle choices, GWAS studies have revealed that genetic polymorphisms are major contributors to osteoporosis.^5–8^ The deterioration in bone microarchitecture and decrease in bone mineral density (BMD) associated with osteoporosis results from abnormal bone remodeling, where there is a net increase in bone resorption when compared to bone formation. Bone remodeling is a process maintained by two key effector cells, bone-building osteoblasts (OBs) and bone-resorbing osteoclasts (OCs). Bone forming OBs originate from multipotent mesenchymal stem cells (MSCs), which also give rise to multiple other cell types including adipocytes, chondrocytes, myocytes, and fibroblasts.^9^ Within the OB lineage, runt-related transcription factor 2 (RUNX2) is perceived as the master regulator of OB differentiation and function. Multiple signaling pathways converge on and regulate RUNX2 activity, including the (1) TGF-β/BMP and (2) WNT pathways, ultimately regulating OB formation and activity.^10,11^ The only known bone-resorbing cells in the body, OCs are terminally differentiated cells of the hematopoietic hierarchy formed from myeloid precursor (MP) cells present in bone. Osteoblasts and other cells present within the bone microenvironment regulate OC activity primarily through secreting the cytokines colony-stimulating factor 1 (CSF1) and receptor activator of Nf-κB ligand (RANKL). These cytokines activate signaling pathways in MPs that result in the increased expression of multiple transcription factors (TFs) essential for subsequent OC differentiation—PU.1, MITF, NFATc1, cFOS, FOSL2, and NF-κB.^12–17^

The Ets family TF, PU.1, is the master regulator of the commitment of hematopoietic precursors to the myeloid lineage^18^ and it has been shown in mouse models to be essential for MP commitment towards OC differentiation.^12^ A known co-partner of PU.1 in OCs is microphthalmia-associated TF (MITF).^19^ PU.1 and MITF physically interact and have been shown to regulate the transcription of multiple genes necessary for OC function including *Acp5*, *Ctsk*, *Oscar* and *Clcn7*.^19–22^ Mice bearing various spontaneous MITF mutations support its requirement for proper osteoclastogenesis.^13,23,24^ Although the interplay between PU.1 and MITF in regulating key effector genes required for OC function is well known, the extent by which PU.1 and MITF affect the full course of OC differentiation is largely unknown.

In this study, we investigated whether known BMD GWASs show an enrichment of SNPs affecting lineage-specific pathways and functions in OC differentiation. This was performed by overlaying SNPs identified in men and women that are associated with decreased BMD (BMD-SNPs) onto active enhancers in the myeloid compartment that are associated with PU.1 binding. We also conducted this analysis in the mesenchymal compartment using RUNX2-associated enhancers as a control group. This process segregated out discrete, lineage-specific molecular functions and biological processes important for OC and OB differentiation. In the myeloid compartment, functional enrichment analysis identified a network of PU.1-regulated TFs in both MPs and OCs. We subsequently used mouse models to show, for the first time, that conditional deletion of *Pu.1* in differentiating OCs results in severe osteopetrosis. However, when PU.1 is knocked down in MPs, there is an increase in osteoclast precursors (OCPs) that also fail to undergo OC differentiation. This phenotype results from a dysregulation of the OC TF network, which we demonstrate using functional genomic analysis of MITF- and PU.1-bound loci. Further, we show that TFs which govern terminal OC differentiation, including *Nfatc1* and *Fosl2*, are regulated by superenhancers cooperatively bound by PU.1 and the BET protein, BRD4. Finally, we identified a conserved PU.1-bound enhancer region in the first intron of murine *Nfatc1*, which requires PU.1 for conformational changes that bring distal enhancers closer to the transcription start site (TSS) of *Nfatc1* by means of chromatin looping.

In summary, by utilizing disease-associated SNPs identified by GWAS in combination with functional genomics and mouse modeling, we have provided critical mechanistic insights into a complex TF network governing OC differentiation. This study highlights the importance and utility of employing large GWAS datasets as a filter to identify lineage-specific functions associated with a polygenic trait, such as osteoporosis.

## Results

### Meta-analysis of BMD loci predicts cell lineage-specific functions and pathways

**Fig. 1:**
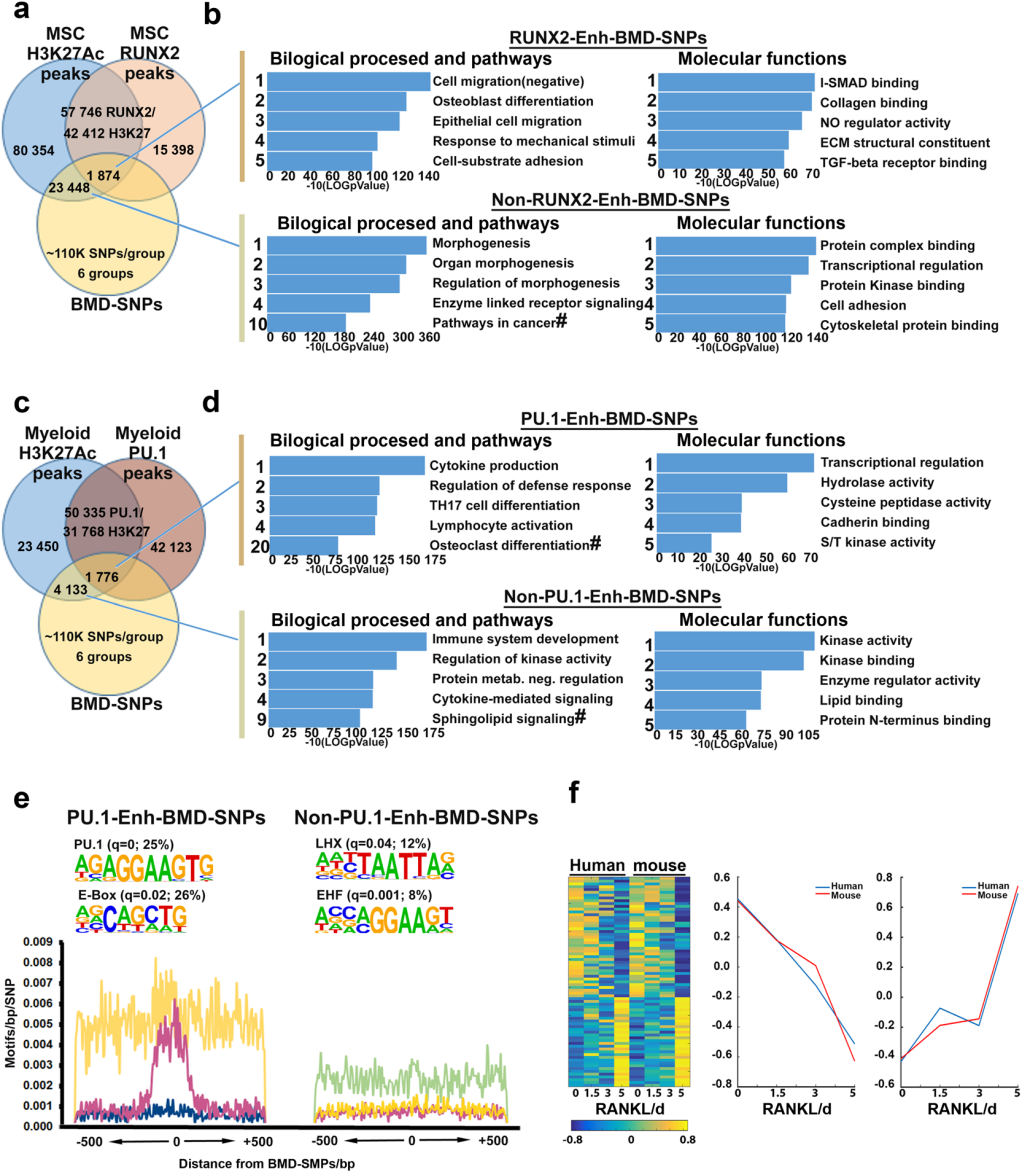
Meta-analysis of BMD loci predicts cell lineage-specific functions and pathways. **a** Venn diagram depicting the overlap of H3K27Ac and RUNX2 ChIP-Seq peak data from human-induced mesenchymal stem cells (iMSCs) with bone mineral density (BMD)-associated SNPs (BMD-SNPs). **b** Top biological processes and molecular functions identified by functional enrichment analysis of RUNX2-Enh-BMD-SNPs (top panel) and Non-RUNX2-Enh-BMD-SNPs (bottom panel). # indicates the top ranking Kegg pathways. **c** Venn diagram depicting the overlap of H3K27Ac and PU.1 ChIP-Seq peaks from human peripheral blood monocytes and macrophages with BMD-SNPs. **d** Top biological processes and molecular functions identified by functional enrichment analysis of PU.1-Enh-BMD-SNPs (top panel) and Non-PU.1-Enh-BMD-SNPs (bottom panel). # indicates the top ranking Kegg pathways. **e** Motifs enriched in the 500 base pair (bp) regions adjacent to PU.1-Enh-BMD-SNPs and Non-PU.1-Enh-BMD-SNPs. For PU.1-Enh-BMD-SNPs, the purple plot represents PU.1, yellow—MITF, and blue—RUNX2 (negative control). For Non-PU.1-Enh-BMD-SNPs, the purple plot also represents PU.1, yellow—EHF, and green—LHX. **f** Orthologous temporal expression of 75 transcription factors that follow similar expression kinetics during mouse and human OC differentiation.

We implemented a pipeline aiming to identify lineage-specific pathways and functions utilizing the GEFOS BMD-SNP database and publically available chromatin immunoprecipitation coupled with high-throughput sequencing (ChIP-Seq) data on the mesenchymal and myeloid lineage-specific TFs, RUNX2, and PU.1, respectively. We overlapped RUNX2 ChIP-Seq data from human-induced MSC (iMSCs),^25^ that were not stimulated to undergo differentiation into more mature osteoblasts, with histone H3 lysine 27 acetylation (H3K27Ac) ChIP-Seq data to mark active enhancers in these cells. The overlap of 73144 RUNX2-bound ChIP-Seq peaks with 122766 H3K27Ac-associated regions denoted 42412 enhancers containing a total of 57746 RUNX2 peaks (Fig. 1a). A similar strategy was performed using PU.1 and H3K27Ac ChIP-Seq data from human peripheral blood monocytes and macrophages.^26^ Overlap of 92458 PU.1 peaks with 55218 H3K27Ac-associated regions resulted in 31768 unique enhancers that overlapped with at least one PU.1 peak (Fig. 1c). We then filtered the RUNX2- or PU.1-bound enhancers as well as non-bound enhancers by the presence of six preprocessed groups (*P* ≤ 0.05) of BMD-SNPs from the GEFOS consortium.^27^ The six groups included in the GEFOS database were femoral neck or lumbar spine BMD-associated SNPs from men, women, or pooled samples. The 42412 RUNX2-bound enhancers contained a total of 1874 unique BMD-SNPs (RUNX2-Enh-BMD-SNPs; Fig. 1a, Supplemental Table 1) and the 80354 enhancers not bound by RUNX2 encompassed 23448 unique BMD-SNPs (Non-RUNX2-Enh-BMD-SNPs; Fig. 1a) from these six groups. Annotation of the genes associated with these loci was done by nearest neighbor analysis and subsequent functional enrichment was performed using Metascape.^28^ Functional enrichment analysis of the RUNX2-Enh-BMD-SNP genes revealed OB differentiation and negative regulation of cell migration as the top ranking biological processes as well as I-SMAD binding as the top molecular function (Fig. 1b, top panel). Non-RUNX2-Enh-BMD-SNPs did not reveal any known OB-specific biological processes and pathways or molecular functions (Fig. 1b, bottom panel). Similarly, the 1776 unique genes associated with PU.1-bound enhancers containing BMD-SNPs (PU.1-Enh-BMD-SNPs; Fig. 1c, Supplemental Table 1) were enriched for OC differentiation as the top KEGG pathway and TF binding as the top molecular function (Fig. 1d, top panel). The molecular functions and biological processes enriched for genes associated with BMD-SNPs in H3K7Ac-enriched enhancer regions not bound by PU.1 (Non-PU.1-Enh-BMD-SNPs) were not as specific to OC differentiation and overall more general in nature than the processes and functions enriched in PU.1-Enh-BMD-SNPs (Fig. 1d, bottom panel).

On the basis of the fact that we used MSCs and the mesenchymal compartment as a control, we focused on the enriched TF binding motifs that are present within 50 bp around PU.1-Enh-BMD-SNPs, which revealed PU.1 and E-box motifs (Fig. 1e, top panel; left). When these motifs were mapped in the 500 bp regions flanking the BMD-SNPs, they congregated within 150 bp around the PU.1-Enh-BMD-SNPs (Fig. 1e bottom left panel). Motif analysis around Non-PU.1-Enh-BMD-SNPs revealed enrichment of LHX and EHF motifs (Fig. 1e, top panel; right). These motifs do not bind TFs with any known function in OC differentiation. Additionally, these motifs did not show a higher occurrence around the SNPs like the motifs around the PU.1-Enh-BMD-SNPs (Fig. 1e, compare bottom left to bottom right panel). Additionally, genes associated with PU.1 peaks found within 500 bp of a BMD-SNP were used for functional enrichment analysis. This analysis also demonstrated TFs and OC differentiation as the major functionally enriched processes in PU.1-associated BMD-SNPs (Supplementary Figure 1A, B). Furthermore, Gene Set Enrichment Analysis (GSEA) from human and mouse OC differentiation revealed TF activity as one of the top enriched gene sets (Supplementary Figure 1C, D). To examine the conservation of the expression of these TFs between species, we compared the temporal expression of TFs obtained from the enrichment analysis between mouse and human OC differentiation using microarray analysis. From this, we found that 75 TFs demonstrated similar expression kinetics between species, including the OC lineage-determining TFs *Nfatc1*, *Fos*, and *Fosl2* (Fig. 1f).

### PU.1 is essential for both osteoclast lineage commitment and differentiation

In silico analysis of the myeloid compartment in human datasets suggested that PU.1 orchestrates a TF network essential for OC differentiation. It has been previously shown that global deletion of *Pu.1* in mice results in early perinatal lethality and loss of myeloid lineages, including OCs.^12^ To determine the effects of *Pu.1* deletion specifically in committed OCPs in vivo, we combined an OC-specific *Cathepsin K Cre* knock-in allele (*CtskCre*) with a floxed *Pu.1* conditional allele (*Pu.1*^*fl*^) to create PU.1 knockdown in differentiating OCs (*Pu.1*^*ΔOC/ΔOC*^). *CtskCre*-mediated recombination of the *Pu.1*^*fl/fl*^ allele was not detected in isolated MPs, but only after they became committed OCs following RANKL treatment in vitro (Supplemental Figure 2A). Examination of protein expression during OC differentiation using this model confirmed that PU.1 expression was progressively lost over time following RANKL treatment (Supplemental Figure 2B).

**Fig. 2:**
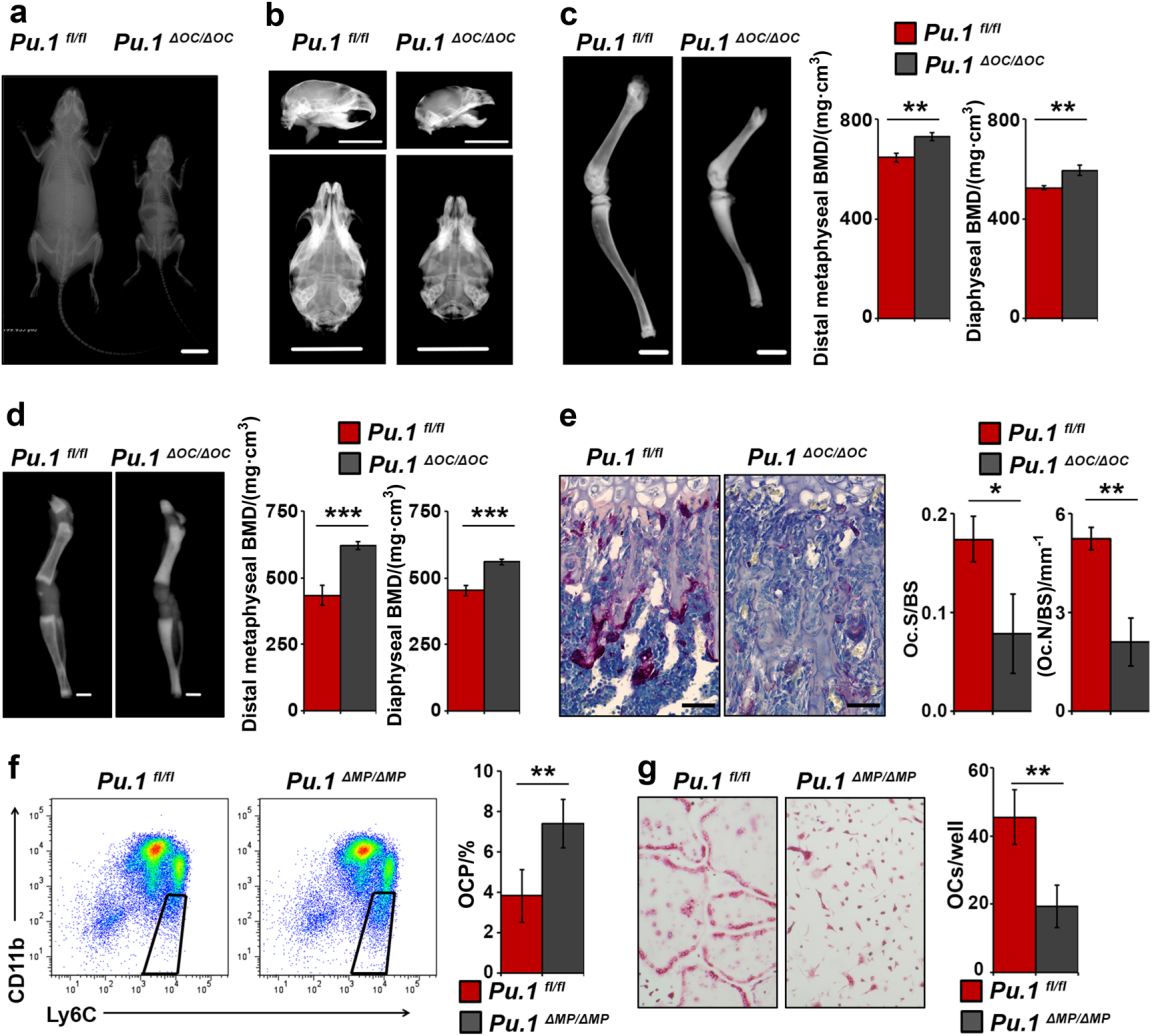
PU.1 is essential for both OC lineage commitment and differentiation. **a** Dorsoventral whole body radiographs of 4-week-old male littermate *Pu.1fl/fl*;*CtskCre*+ (*Pu.1ΔOC/ΔOC*) mice and *Pu.1fl/fl* controls. Image is representative of *n* = 3 pairs. Scale bar = 1 cm. **b** Lateral (top) and dorsoventral (bottom) radiographs of the skulls of 4-week-old littermate *Pu.1ΔOC/ΔOC* mice and *Pu.1fl/fl* controls. Image is representative of *n* = 3 pairs. Scale bars = 1 cm. **c** Lateral femoral and tibial radiographs and bone mineral density (BMD) quantification of the distal metaphyseal and diaphyseal regions of the femurs of 4-week-old littermate *Pu.1ΔOC/ΔOC* mice and *Pu.1fl/fl* controls (*n* = 3). Scale bars = 2.5 mm. **d** Same as **c** except from 8-day-old littermate *Pu.1ΔOC/ΔOC* mice and *Pu.1fl/fl* controls (*n* = 4 *Pu.1fl/fl*, *n* = 3 *Pu.1ΔOC/ΔOC*). Scale bars = 1 mm. **e** Histologic images and histomorphometry of the distal femoral metaphyses of the mice in **d** (*n* = 3). Scale bars = 50 μm. **f** Flow plots and quantification of CD11b-/loLy6Chi osteoclast precursors (OCPs) in the BM of 6–8-week-old *Pu.1ΔMP/ΔMP* mice and *Pu.1fl/fl* controls (*n* = 4). **g** Images and quantification of TRAP staining of in vitro differentiated OCPs from **f** (*n* = 4). For all figures and subfigures, data are represented as the mean ± S.D. for all bar and line graphs and all images are representative. In addition, for all figures and subfigures, *P < 0.05, **P < 0.01, ***P < 0.001, and ****P < 0.0001.

In vivo analysis revealed that *Pu.1*^*ΔOC/ΔOC*^ mice exhibited severe growth and skeletal defects at four weeks of age compared to controls (Fig. 2a-c, Supplemental Figure 2C, E). In addition, *Pu.1*^*ΔOC/ΔOC*^ mice did not display tooth eruption at this time point (Fig. 2b, Supplemental Figure 2E). Digital radiographic analysis of the femurs of these mice showed a 13% increase in BMD over controls in both the distal metaphysis and the diaphysis (Fig. 2c). Similarly, digital radiographic analysis of the femurs of 8 day old *Pu.1*^*ΔOC/ΔOC*^ mice revealed a 25% increase in BMD in the diaphysis and a 36% increase in the distal metaphysis compared to controls (Fig. 2d and Supplemental Figure 2D). Analysis of TRAP-stained femurs revealed severe OC defects, with a threefold decrease in both OC surface to bone surface (Oc.S/BS) and OC number to bone surface (Oc.N/BS) (Fig. 2e). Consistent with this in vivo phenotype, in vitro differentiation of MPs from *Pu.1*^*ΔOC/ΔOC*^ mice formed nearly six-fold fewer mature, multinucleated TRAP + OCs than controls (Supplemental Figure 2F).

To determine the effects of myeloid-specific PU.1 deletion on commitment to the OC lineage, we selected a tamoxifen-inducible, myeloid-lineage-specific *Csf1rTAMCre* allele. When combined with *Pu.1*^*fl/fl*^ mice, we generated inducible *Pu.1*^*ΔMP/ΔMP*^ mice (Supplemental Figure 2G–I). Using this model, we examined the effects of PU.1 knockdown in MPs initially by analyzing their more differentiated descendants in the bone marrow (BM) – granulocytes (PMNs), pro-inflammatory monocytes (PIMs), common monocyte dendritic precursors (MDPs), and osteoclast precursors (OCPs)^29^ by flow cytometry. Unexpectedly, there was over a twofold increase in OCPs in *Pu.1*^*ΔMP/ΔMP*^ mice compared to controls (Fig. 2f). A similar increase in OCPs was also observed in the peripheral blood of *Pu.1*^*ΔMP/ΔMP*^ mice (Supplemental Figure 2J). Deletion of *Pu.1* in MPs had no effect on the percentage of other myeloid descendants in the BM, which were identified by cell surface markers (Supplemental Figure 2K). Interestingly, when BM OCPs isolated by fluorescence activated cell sorting (FACS) were cultured with CSF1/RANKL to induce OC differentiation, *Pu.1*^*ΔMP/ΔMP*^ mice formed 2.4-fold fewer OCs than control mice (Fig. 2g). The effect of PU.1 deletion on the functionality of the other myeloid-derived cell populations was not assessed. These findings suggest that *Pu.1* deletion does not affect OCP formation but rather reduces their differentiation potential.

### PU.1 is associated with *cis*-acting elements critical for osteoclast differentiation and bone remodeling irrespective of RANKL signaling

**Fig. 3:**
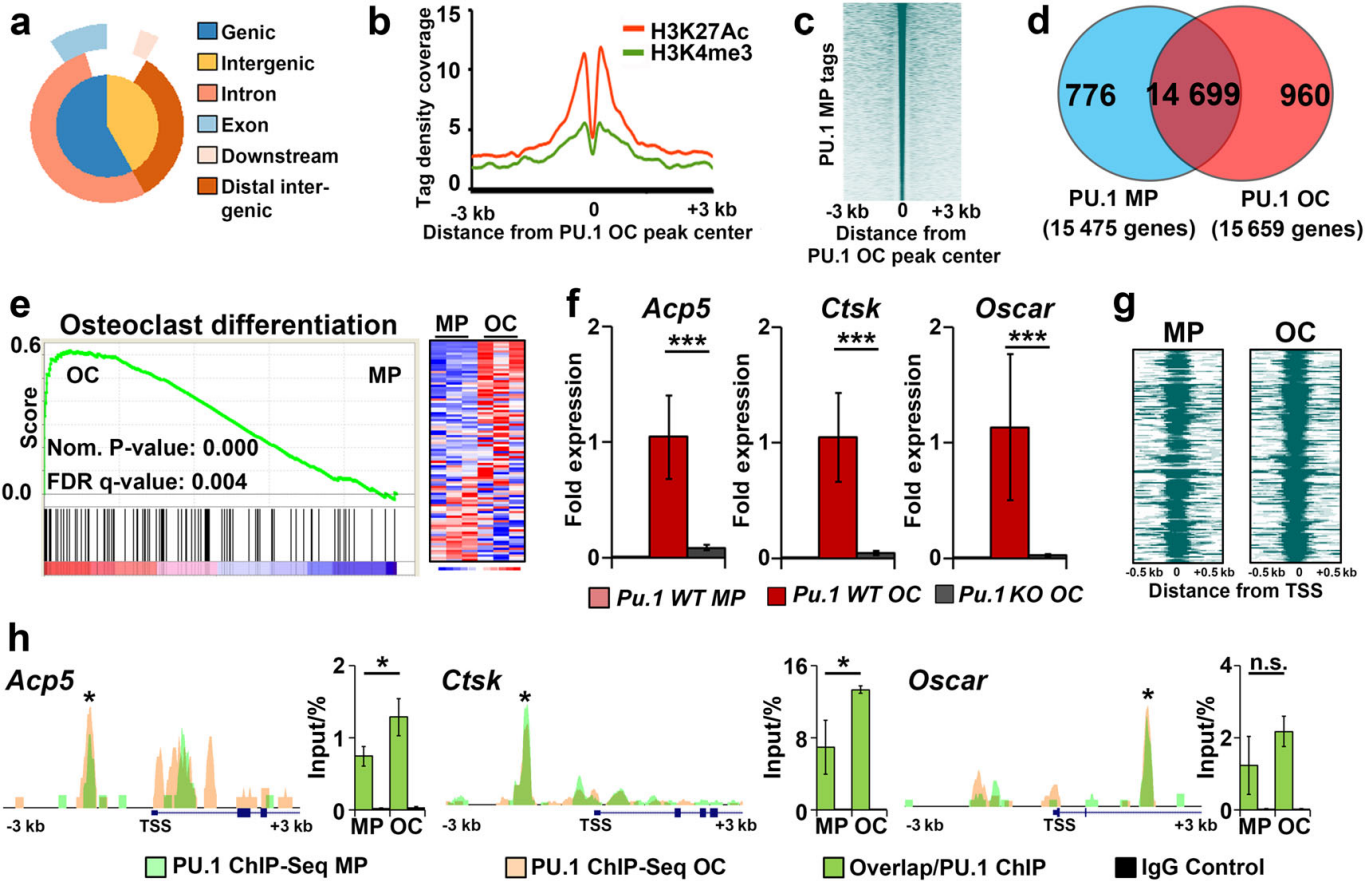
PU.1 is associated with *cis*-acting elements critical for OC differentiation and bone remodeling irrespective of RANKL signaling. **a** Graphical representation of the distribution of PU.1 OC ChIP-Seq peaks throughout the mouse genome. **b** ChIP-Seq tag density coverage of murine BMDM H3K27Ac active enhancer marks (red) and murine BMDM H3K4me3 active promoter marks (green) ± 3 kilobases (kb) from PU.1 OC peak centers. **c** Treeview plot of genome-wide PU.1 myeloid precursor (MP) tags ± 3 kb from PU.1 OC peak centers. **d** Venn diagram depicting the number of genes in MPs and OCs with PU.1 peaks. **e** GSEA plot of an OC differentiation gene set significantly enriched in genes with PU.1 OC peaks using our murine MP and OC microarray data (*n* = 3). Heatmap (right) indicating MP and OC expression of genes in the gene list. **f** RT-qPCR analysis of genes on the GSEA gene list essential for OC function. Gene expression is shown for wild-type (WT) MPs and OCs and *Pu.1 KO* OCs (*n* = 3). **g** Treeview plot of PU.1 MP and OC tags ± 500 base pairs from the transcriptional start sites (TSS) of all genes on the GSEA list. **h** Depiction of MP and OC PU.1 ChIP-Seq peaks ± 3 kb from the TSS of *Acp5*, *Ctsk*, and *Oscar*. Conventional ChIP validation of PU.1 binding to the starred sites is shown (bar graphs, *n* = 3).

To address the molecular functions of PU.1 in OC differentiation, the genomic location of PU.1 binding sites was determined by ChIP-Seq in both murine MPs and OCs. Mapping ChIP-Seq reads to the mouse genome revealed ~77000 loci enriched for PU.1 binding in OCs (Supplemental Table 2). These PU.1-bound loci were dispersed throughout the genome and were most frequently found in distal regions located 10–100 kb from known TSSs in intergenic regions or within gene introns (Fig. 3a, Supplemental Figure 3A). Further, PU.1 OC peaks were enriched for H3K27Ac, a marker of active enhancers, when compared with publically available histone mark ChIP-Seq data from murine myeloid cells (Fig. 3b).^30,31^ In contrast, there was less enrichment of H3K4me3, a proximal promoter marker, near PU.1 OC peaks (Fig. 3b).^32^

ChIP-Seq was also performed on murine MPs. Interestingly, the majority of PU.1 peaks were shared between OCs and MPs as demonstrated by the PU.1 sequence tag densities in MPs located around PU.1 peak centers in OCs (Fig. 3c). This indicates that PU.1 binds to similar loci even before differentiation is initiated. When genes associated with PU.1 peaks in MPs and OCs were compared, ~90% were shared between the two (Fig. 3d).

In the next layer of analysis, PU.1 OC ChIP-Seq data was overlaid with global gene expression profiling performed in murine MPs and OCs. GSEA demonstrated that “Osteoclast Differentiation” and “Bone Remodeling and Resorption” were the top processes significantly enriched in genes bound by PU.1 in OCs (Fig. 3e and Supplemental Figure 3B, Supplemental Table 3). Gene expression analysis by RT-qPCR verified that these genes were upregulated in OCs compared to MPs and comparison of wild-type OCs to *Pu.1 KO* OCs confirmed that upregulation of these genes was PU.1-dependent (Fig. 3f, Supplemental Figure 3C). As in the genome-wide analysis, the majority of these differentially regulated genes displayed PU.1 occupancy in both MPs and OCs (Fig. 3g, Supplemental Figure 3D). Conventional ChIP confirmed that PU.1 was bound to regions proximal to the TSS of 10 representative target genes identified by ChIP-Seq (Fig. 3h and Supplemental Figure 3E).

### PU.1 and its co-partner MITF regulate the expression of a network of TFs necessary for OC differentiation

**Fig. 4:**
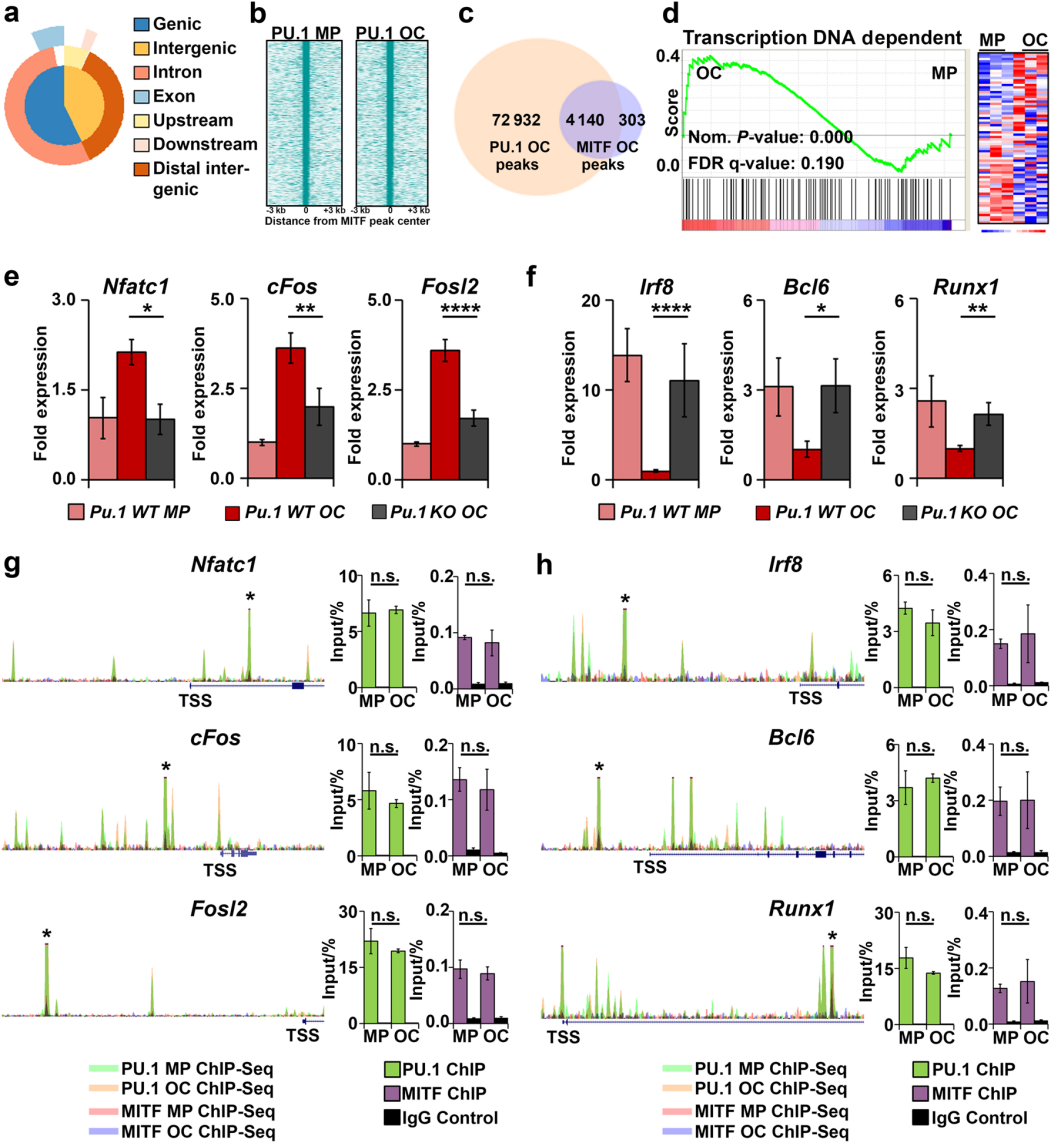
PU.1 and its co-partner MITF regulate the expression of a network of TFs necessary for OC differentiation. **a** Graphical representation of the distribution of MITF OC ChIP-Seq peaks throughout the genome. **b** Treeview plot of genome-wide PU.1 MP and PU.1 OC ChIP-Seq tags ± 3 000 base pairs from MITF MP or MITF OC peak centers. **c** Venn diagram depicting the OC ChIP-Seq peak sites shared between PU.1 and MITF. **d** GSEA plot of a TF gene set significantly enriched in genes with overlapping PU.1 and MITF OC ChIP-Seq peaks using our murine MP and OC microarray data (*n* = 3). Heatmap (right) indicating MP and OC expression of genes in the gene list. **e** RT-qPCR analysis of 3 TFs on the GSEA gene list, which are necessary for OC differentiation. Gene expression is shown for WT MPs and OCs and *Pu.1 KO* OCs (*n* = 3). **f** RT-qPCR analysis of 3 TFs on the GSEA gene list which inhibit OC differentiation. Gene expression is shown for WT MPs and OCs and *Pu.1 KO* OCs (*n* = 3). **g**, **h** Depiction of MP and OC PU.1 and MITF ChIP-Seq peaks near the TF loci analyzed in **e** and **f**. Each trace is 30 kb wide and the TSS is indicated. Conventional ChIP validation of PU.1 and MITF binding to the starred sites is shown (bar graphs, *n* = 3).

The majority of BMD-SNPs found in PU.1/H3K27Ac marked enhancers showed an increased occurrence of PU.1 and MITF (E-box) motifs near the SNP sites (Fig. 1e). Further, we have previously shown in OCs that PU.1 and its co-partner MITF regulate genes essential for OC function.^19,20^ MITF ChIP-Seq analysis in MPs and OCs indicated that the majority of MITF peaks were distributed throughout the genome primarily in intergenic and intronic sequences located 10–100 kb from genes, similar to PU.1 (Fig. 4a, Supplemental Figure 4A, Supplemental Table 2). PU.1 sequence tags from individual MP and OC ChIP-Seq data were significantly enriched at the center of MITF-bound loci in MPs and OCs, respectively (Fig. 4b). Even though only ~5% of PU.1 peaks in OCs were bound by MITF, this comprised >90% of MITF-bound loci in OCs (Fig. 4c). Overlap of the 4140 PU.1/MITF co-bound peaks with global gene expression once again revealed OC differentiation as a significantly enriched process (Supplemental Figure 4B, Supplemental Table 3). Similar to the human functional genomic analysis (Fig. 1d), target genes of PU.1/MITF were also enriched for TFs involved in DNA-dependent transcription (Fig. 4d, Supplemental Table 3).

To validate these ChIP-Seq peaks, we focused on a set of TFs with both known functions in OC differentiation and a PU.1/MITF peak located within 35 kb of the gene’s TSS. The results of this analysis confirmed that key TFs that promote (*Nfatc1*,^14^ *Fosl2*,^17^ and *cFos*^15^) or inhibit (*Irf8*,^33^ *Bcl6*,^34^ and *Runx1*^35^) OC differentiation were targets of both PU.1 and MITF (Fig. 4e–h). In total, we have validated PU.1 and MITF binding to 18 TFs with established functions in OC differentiation (Fig. 4g, h and Supplemental Figure 4C). These results indicate that PU.1 regulates OC differentiation by means of promoting the expression of downstream pro-osteoclastogenic TFs and suppressing the expression of TFs that negatively regulate OC differentiation.

### Interdependent recruitment of both PU.1 and BRD4 marks superenhancers required for OC differentiation

**Fig. 5:**
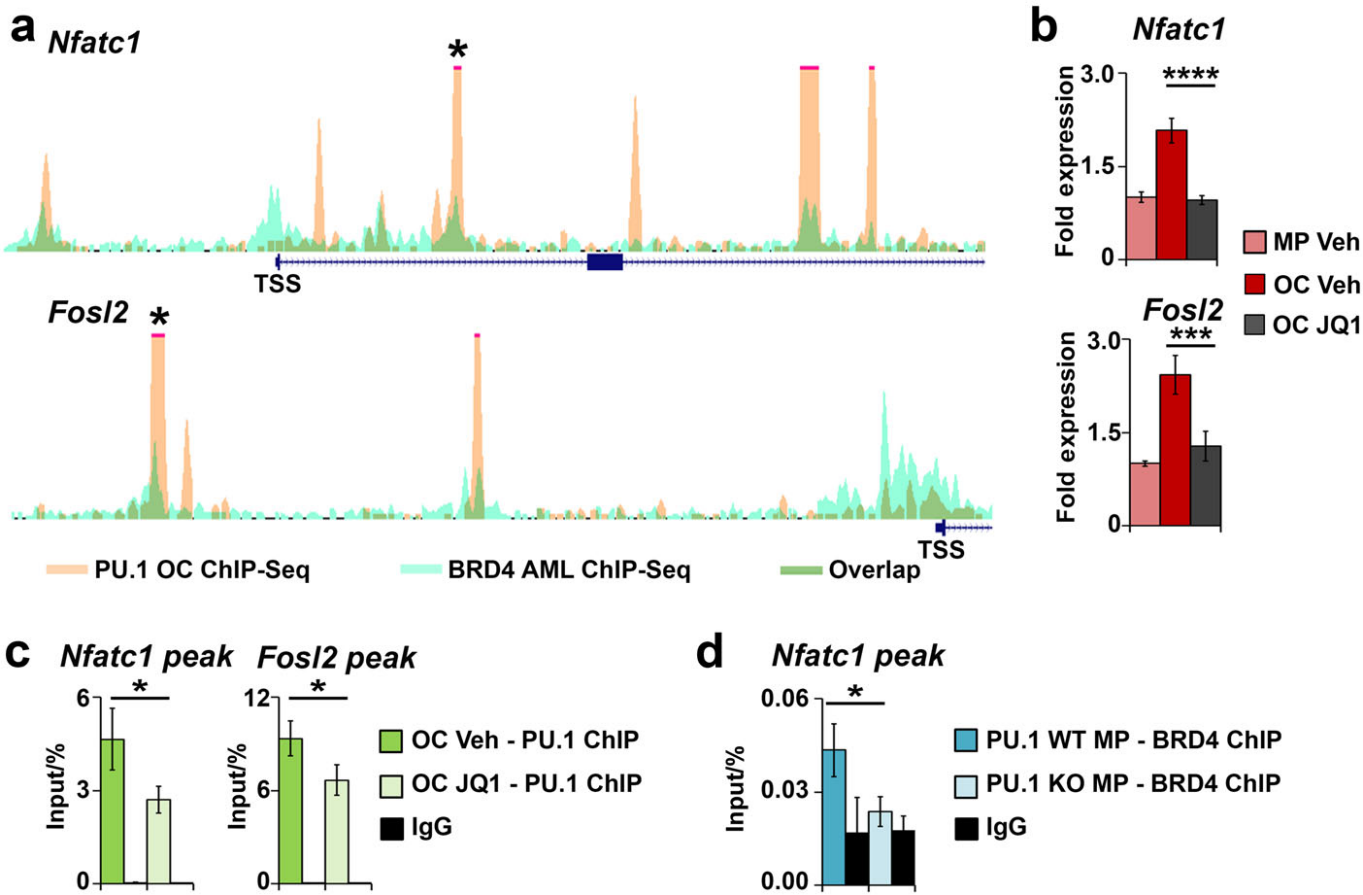
Interdependent recruitment of both PU.1 and BRD4 marks superenhancers required for OC differentiation. **a** Depiction of PU.1 OC ChIP-Seq peak overlap with BRD4 ChIP-Seq data from murine acute myeloid leukemia (AML) cells. Each trace is 30 kb wide and the TSS is located as indicated. **b** RT-qPCR analysis of the expression of OC TF network genes, *Nfatc1* and *Fosl2* in response to treatment with the BRD4 inhibitor JQ1 (*n* = 3). **c** Conventional ChIP of PU.1 binding to peak sites starred in **a** in OCs with and without JQ1 treatment (*n* = 3). **d** Conventional ChIP of BRD4 binding to the *Nfatc1* peak site starred in **a** in WT and *Pu.1 KO* MPs (*n* = 3).

More in-depth analysis of the DNA-binding pattern of PU.1 in OCs uncovered that clusters of PU.1 peaks throughout the genome formed nearly 700 superenhancers (Supplemental Table 5). Furthermore, there was significant overlap between the PU.1 ChIP-Seq peaks which form superenhancers in OCs and publically available BRD4 ChIP-Seq data from murine acute myeloid leukemia (AML) cells (Supplemental Figure 5A).^36^ Several of these superenhancer loci were situated at lineage determinant TFs important for OC differentiation such as *Nfatc1* and *Fosl2*, which displayed strong overlap of PU.1 peaks with publically available BRD4 AML ChIP-Seq peaks (Fig. 5a). We utilized JQ1, a BRD4 bromodomain inhibitor, to determine the effects of blocking superenhancer-mediated gene transcription on the expression of PU.1 target TFs in OCs.^37^ This analysis confirmed that expression of both *Nfatc1* and *Fosl2* in OCs was inhibited by JQ1 (Fig. 5b). In addition, JQ1 significantly reduced PU.1 enrichment at PU.1 enhancers near *Nfatc1* and *Fosl2* (Fig. 5c). Moreover, using wild-type and *Pu.1 KO* cells, we observed decreased PU.1 enrichment at one of the *Nfatc1* peaks in *Pu.1 KO* cells, which correlated with a concomitant decrease in BRD4 enrichment at the same site (Fig. 5d and Supplemental Figure 5B).

### PU.1 is necessary for looping of a conserved enhancer at the Nfatc1 locus

**Fig. 6:**
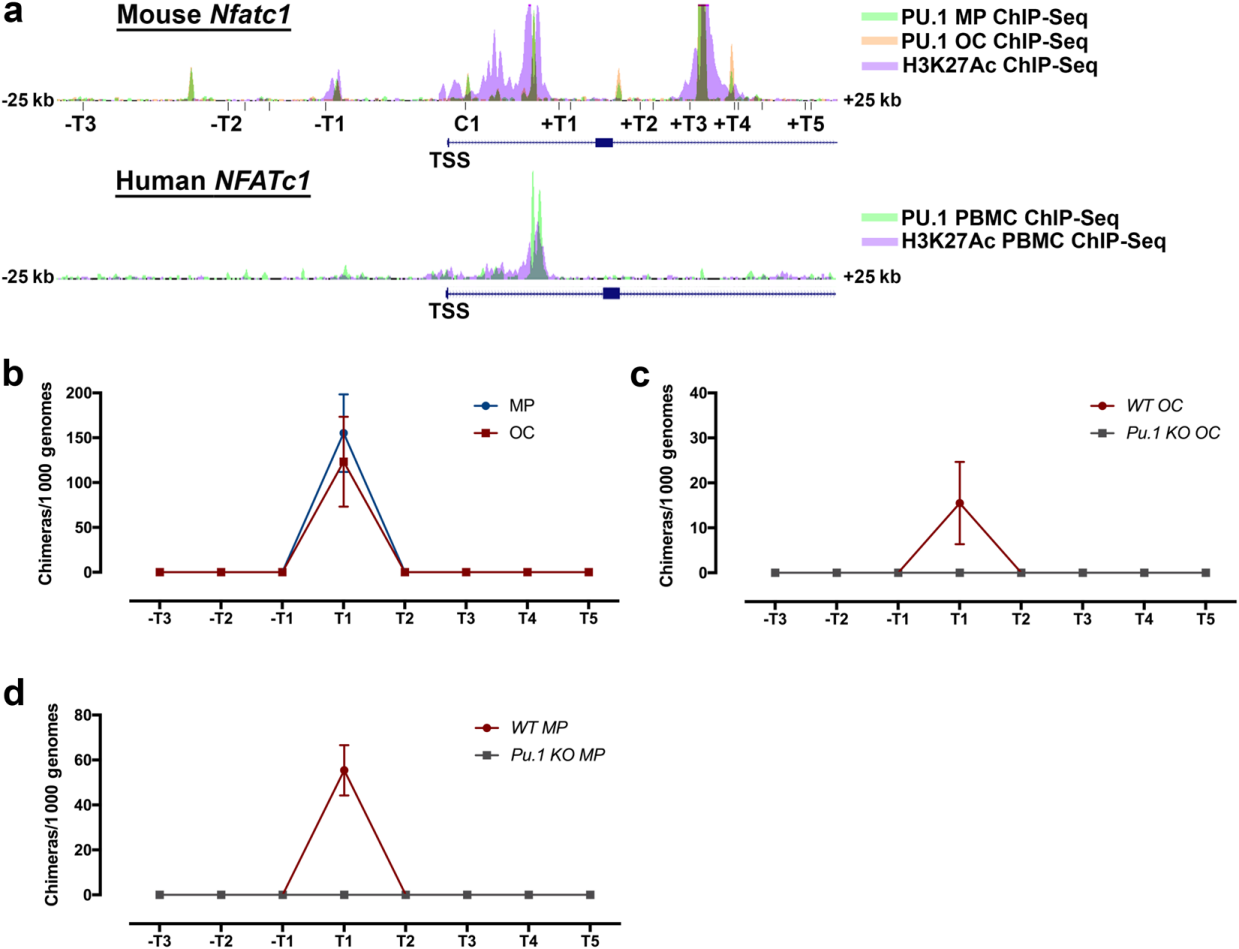
PU.1 is necessary for looping a conserved enhancer at the Nfatc1 locus. **a** Depiction of murine PU.1 MP and OC ChIP-Seq peaks and murine BMDM H3K27Ac active enhancer mark ChIP-Seq peaks ± 25 kb from the TSS of *Nfatc1* (top). *Bgl*II sites used for 3C (below top trace). Trace of PU.1 and H3K27Ac ChIP-Seq peaks from human PBMCs ± 25 kb from the TSS of *NFATc1* (bottom). **b** 3C assay from murine MPs and OCs (*n* = 2). *Bgl*II sites on the *x*-axis correspond to the indicated site ligating with the common forward site (C1) at the *Nfatc1* promoter. **c** 3C assay results from murine WT OCs and *Pu.1 KO* OCs (*n* = 2). **d** 3C assay from murine WT MPs and *Pu.1 KO* MPs (*n* = 2).

To evaluate the functional relevance of the distal enhancers that are enriched for PU.1 in both MPs and OCs, we used a chromosome conformation capture (3C) assay. This assay allows us to investigate the relationship between chromatin folding and transcriptional activity,^38^ of which we used the OC master regulator *Nfatc1* as a candidate gene. In both murine OCs and MPs, the *Nfatc1* locus has two intronic enhancers, both marked by H3K27Ac, which overlap PU.1 peaks (Fig. 6a). Comparison to PU.1-bound sites in human monocytes^26^ indicated that only the enhancer in intron 1 was conserved (Fig. 6a). Our 3C assay revealed a looping event between the PU.1/MITF-bound peak site in intron 1 (marked T1) and the *Nfatc1* promoter (marked C1, Fig. 6a). Of the 8 distal loci tested, this enhancer present in intron 1 was the only site of chromatin looping (Fig. 6b). This looping event occurred similarly in MPs and OCs, indicating that loop formation is independent of RANKL signaling (Fig. 6b). Importantly, this intron 1 looping event did not occur in either OCs or MPs lacking PU.1 (Fig. 6c, d, respectively).

## Discussion

Mining ChIP-Seq data for physiologically relevant targets has utilized many strategies for functional enrichment. Owing to the large number of gene targets obtained in a ChIP-Seq experiment, organizational strategies such as (1) determining the presence or absence of motifs; (2) assessment of overlap with other TF binding regions; and (3) evaluation of differential binding are used as strategies to filter and preprocess the gene list. However, integrating ChIP-Seq data with data obtained from GWAS analysis has also been used to better understand the functionality and biological relevance of SNPs present in non-coding regions. Some of the strategies used include (1) functional enrichment analysis of SNP-associated genes; (2) motif analysis of SNP loci; and (3) integration with ChIP-Seq data from epigenetic modifiers and TFs.^26,39–41^ Interestingly, SNPs or variants enriched in lineage-specific enhancer regions have been shown to reflect the phenotype affecting the cell type.^26,42^ In the present study, by using BMD-SNPs from the GEFOS dataset^27^ as a limiting factor to enrich functional groups in both myeloid^26^ and MSC enhancers,^25^ we are able to elucidate lineage-specific physiologically relevant pathways and functions. In contrast, non-filtered enhancer-associated genes that overlapped with BMD-SNPs as well as lineage-specific enhancers that did not overlap with BMD-SNPs failed to enrich for known cell type-specific pathways (data not shown). Interestingly, the majority of TFs from our systems biology approach had similar expression kinetics during mouse and human OC differentiation, enabling and justifying our use of model organisms to test our hypothesis.

PU.1 is exclusively expressed in cells of the hematopoietic lineage. Dynamic fluctuation in its expression dictates differentiation in a dose-dependent manner toward multiple cell fates.^43^ This occurs by PU.1 interacting with, and regulating the expression of, multiple lineage determining and lineage function genes. During commitment to a particular cell lineage, PU.1 is highly expressed in myeloid cells, present in a lower, but still very important level in B lymphocytes, and even further downregulated in T and erythroid cells.^44^ With specific regard to myeloid cells, sustained high PU.1 levels favor macrophage over granulocyte development. When combined with the fact that PU.1 regulates multiple myeloid-specific genes during differentiation, PU.1 is considered to be the master regulator of myeloid development.^43,45^ Multiple mouse models of PU.1 mutations or deletions have been reported. Although one model of global PU.1 deletion resulted in late embryonic lethality and the absence of B lymphocytes, macrophages, and granulocytes,^46^ another yet similar mouse model experienced perinatal lethality within 48 h of birth. However, this latter model demonstrated a very small number of mature macrophages and granulocytes in the spleen and BM when mouse lifespan was prolonged with antibiotic therapy.^47^ When combined with a lack of OC formation and resultant osteopetrosis in the latter model, these cumulative findings are consistent with impaired myeloid development.^12^

With the advent of conditional knock-outs, Polli et. al.^48^ demonstrated that B cell-specific PU.1 deletion resulted in a normal number of B cells during all stages of differentiation. Therefore, we were interested whether PU.1 was indeed needed at all stages of OC differentiation. Although decreased OC differentiation following conditional PU.1 deletion in MPs in our study is anticipated based on historical results, our study demonstrates for the first time that PU.1 is also required for OC differentiation after MPs become committed OCPs. We demonstrated this through a reduction in OC differentiation following PU.1 deletion specifically in committed OC precursors using OC-specific *CtskCre*. This result demonstrated that, unlike in B cells, PU.1 is required throughout OC differentiation. Interestingly, both mice with disruption of the PU.1 DNA-binding domain and a more recent model with conditional disruption of PU.1 using the inducible *Mx1Cre* demonstrated inhibited maturation, but not proliferation, with accumulation of an abnormally large population of immature myeloid progenitor cells.^18,47^ Our finding of increased OCPs following MP-specific deletion of PU.1 which fail to differentiate into functional OCs is consistent with this finding. In total, these results illustrate the requirement of PU.1 during the entire course of OC differentiation.

To evaluate the molecular basis of PU.1 regulation in OCs, we performed ChIP-Seq to identify specific PU.1 targets at different stages of OC differentiation. However, PU.1 occupancy was found near the majority of protein coding gene loci irrespective of the stage of osteoclastogenesis. Previously, we have shown that RANKL signaling targets the MITF-PU.1 complex through phosphorylation of MITF at serine 307 by p38 MAP-kinase.^20,49,50^ The phosphorylation switch correlates with the dissociation of the EOS repressor complex and recruitment of Swi/SNF complex to MITF-PU.1 target promoters.^20,51^ Though MITF and PU.1 occupy these promoters irrespective of the stage of differentiation. Similarly, MITF ChIP-Seq in MPs and differentiating OCs demonstrated that like PU.1, MITF occupancy was observed in MPs and OCs alike. Further, substantiating our pre-genomics era hypothesis that PU.1 is the partner of MITF in MPs and OCs and that they genetically and physically interact,^19,20^ the majority of the MITF-bound loci overlapped with PU.1-bound loci. This hypothesis was also supported by our finding of an increased occurrence of the MITF E-box motif near BMD-SNPs in PU.1 enhancers (Fig. 1e). When genes that are regulated by PU.1/MITF-bound loci were overlaid with MP and OC gene expression data, TFs were identified as a major molecular function regulated by the MITF-PU.1 complex. The TFs that were identified by integrated analysis of MITF/PU.1 ChIP-Seq and gene expression analysis fall into two distinct subsets, those that favor OC differentiation, such as *Nfatc1*, *Fosl2*, and *cFos* (Fig. 4e), as well as those that suppress OC differentiation (*Irf8*, *Bcl6*, and *Runx1*) (Fig. 4f). Of note, in PU.1 knockdown cells this regulatory loop is reversed, which favors the suppression of OC differentiation.

Interestingly, the genes encoding several key receptors (*Csf1r/cfms*, *Tnfrsf11a/Rank*), TFs (*Nfatc1*, *cFos*, *Fosl2*) and OC markers required for differentiation and function (for example, *Ctsk*) reside in what are generally referred to as transcriptional hot spots or superenhancers (Supplemental Table 5). These closely-associated strong enhancers were bound by PU.1. Further analysis of two key TFs, *Nfatc1* and *Fosl2*, for the BET protein family member BRD4 revealed that these TF-encoding genes were regulated by superenhancers and exhibiting cooperative binding of PU.1 and BRD4. Recently, the BET inhibitor JQ1 has been shown to inhibit OC differentiation both in vitro and in vivo.^52^ Here we show that PU.1 is cooperatively bound to BRD4 in an interdependent manner, regulating key TFs required for OC differentiation and function. This finding substantiates earlier results implicating superenhancers as cell lineage determinants within the hematopoietic hierarchy.^53^ Our work provides a mechanistic basis for the recent suggestion for the use of BET inhibition to treat diseases associated with excessive OC activity, including osteoporosis and rheumatoid arthritis.^52,54–56^

An interesting feature observed in these superenhancers was that many enhancer loci fall in distal intronic regions or intergenic regions. The presence of PU.1 at these loci irrespective of signaling indicated that PU.1 facilitates chromatin to be in a conformation enabling rapid induction of transcription when it is needed. The role of PU.1 in chromatin conformation has been reported.^57,58^ Our results at the *Nfatc1* locus substantiate the hypothesis that distal intronic enhancer loci loop back to more proximally located promoter locus. Although RANKL signaling had no effect on this looping event, PU.1 was essential in this process. Surprisingly, PU.1-Enh-BMD-SNPs identified by integrating human myeloid lineage ChIP-Seq with GWAS data from the GEFOS reside in this region. However, the nominal *P* values of these SNPs did not reach the significance threshold. These observations dovetail with our hypothesis that disease-associated SNPs in non-coding regions could shed light on important molecular pathways and functions involved in these diseases, even if a specific statistical significance threshold is not reached.

In summary, by combining functional genomics, mouse modeling, and disease-associated SNPs identified by GWAS, we provide critical mechanistic insights into the regulation of a complex TF network governing OC differentiation. Our in vivo, in vitro, and functional genomic analysis reveals that PU.1 and its co-partner, MITF, regulate a set of TFs whose functions are essential for OC differentiation and function. This study serves as a prototype for identifying alleles from a specific cell type which contribute to the pathogenesis of complex human diseases.

## Materials and methods

### Animals and genotyping

All animals were approved by The Ohio State University Institutional Animal Care and Use Committee (Protocols: 2007A0120-R2 and 2016A00000035). All mice were maintained on the C57BL/6J background (F10 or further). C57BL/6J wild-type mice were purchased from Jackson Laboratories (Bar Harbor, ME). *Pu.1*^*fl/fl*^ mice were a kind gift from Dr. Dan Tenen (Harvard Medical School).^18^ Mice possessing the tamoxifen-inducible myeloid lineage-specific *Csf1r* promoter driven *Cre* allele (*Csf1rTAMCre*) were a kind gift from Dr. Jeffrey Pollard (Albert Einstein College of Medicine).^59^ Mice expressing the OC-specific cathepsin K promoter driven *Cre* (*CtskCre*) were a kind gift from Dr. Steven Teitelbaum (Washington University School of Medicine).^60^

### Antibodies and reagents

All antibodies were mouse specific unless otherwise noted. Rabbit anti-mouse PU.1 and MITF antibodies used for ChIP and WB have been described previously.^20^ BRD4 antibody was purchased from Bethyl Laboratories (Montgomery, TX). LAMIN B antibody was purchased from Santa Cruz Biotechnology (Dallas, TX). Anti-rabbit and anti-goat secondary antibodies (LI-COR Biosciences, Lincoln, NE) were used for WB. Tamoxifen and 4-hydroxytamoxifen (4-OHT) were purchased from Sigma-Aldrich (St. Louis, MO). Tamoxifen was dissolved in corn oil with 5% ethanol and 4-OHT was dissolved in 95% ethanol. The bromodomain inhibitor JQ1 was a kind gift from Dr. Jay Bradner (Harvard Medical School).^37^

### Cell culture and in vitro OC differentiation

For in vitro OC differentiation, either primary BM cells or splenocytes were enriched for MPs initially by culturing on non-adherent plastic plates in Dulbecco’s Modified Eagle Medium (DMEM, Gibco, Life Technologies, Carlsbad, CA) supplemented with 10% fetal bovine serum (FBS, Sigma-Aldrich), 50 U·mL^–1^ penicillin/streptomycin (P/S, Gibco) and 50 ng·mL^–1^ recombinant human CSF1 (Peprotech, Rocky Hill, NJ). After 3 days of culture, the myeloid enriched non-adherent fraction was mechanically isolated and replated on adherent tissue culture plates in DMEM containing 10% FBS, 50 U·mL^–1^ P/S, 50 ng·mL^–1^ CSF1, and 100 ng·mL^–1^ recombinant human RANKL (Peprotech) and harvested at the indicated time points. Where indicated, primary BM-derived myeloid cells (BMDM) undergoing in vitro OC differentiation were treated with 250 nmol·L^–1^ JQ1 or equivalent volume of DMSO (Vehicle control) in the culture media. For extraction and culture of *Pu.1 KO* cells derived from *Pu.1*^*fl/null*^ *Csf1rTAMCre* + mice, mice were treated in vivo with 5 daily intraperitoneal (IP) injections of 1 mg tamoxifen/mouse/day. One day following the final injection, BMs were flushed from femurs and tibias and plated on non-adherent plates as described above with the addition of 1 μmol·L^–1^ 4-OHT in the media. When BMDMs were replated on adherent plates for in vitro osteoclast differentiation, 4-OHT was removed from the media.

### Protein isolation and western blotting

Nuclear lysates were prepared using PIPES nuclear isolation buffer (20 mmol·L^–1^ PIPES, 85 mmol·L^–1^ KCl, 0.5% NP-40, 1 mmol·L^–1^ PMSF, supplemented with protease and phosphatase inhibitors (Sigma-Aldrich)). Nuclear pellets were lysed in RIPA buffer (50 mmol·L^–1^ Tris-HCl, 150 mmol·L^–1^ sodium chloride, 1% NP-40, 1% Na-deoxycholate, 0.1% SDS, 1 mmol·L^–1^ PMSF, supplemented with protease and phosphatase inhibitors). Total nuclear protein was quantified using the D_C_ Protein Assay (Bio-Rad, Hercules, CA) and resuspended in Laemmli Sample Buffer (Bio-Rad). Proteins were separated using SDS-PAGE and transferred to nitrocellulose membranes which were then probed with the indicated antibodies and scanned on an Odyssey CLx scanner (LI-COR Biosciences).

### RNA, RT-qPCR, and microarray analysis

Total RNA was isolated from cells with TRIzol (Invitrogen, Life Technologies). For RT-qPCR, RNA was reversed transcribed to cDNA using SuperScript III Reverse Transcriptase (Invitrogen). qPCR was performed using Taqman Master Mix (Roche, Basal, Switzerland) and Universal Probe Library probes and primers (Roche; Supplemental Table 6). Ribosomal *Rpl4* was used as the housekeeping gene and fold expression calculated using the 2^−∆∆Ct^ method.

Orthologous comparison of expression kinetics during human and mouse OC differentiation was performed using Affymetrix HGU133 and Mouse 430 Gene Chips, respectively (Affymetrix, Santa Clara, CA). Briefly, gene symbols were collapsed using the probeset value with highest mean signal. All gene symbols that represent the transcription cluster were collapsed into unique identifiers and their respective expression values were retained. In both human and mouse expression data only the genes that follow similar expression kinetics were retained and clustered over eight samples using centered *K*-means clustering.

Global analysis of differentially expressed transcriptome profiles between mouse OCs and MPs was performed using the Affymetrix Gene Chip Mouse Exon 1.0_ST (Affymetrix) using the Affymetrix Expression Console software, GSEA v2.0 was used to determine the enrichment of the C5 categories within the Molecular Signatures Database (MSigDB). Statistical significance in each case was determined using 1000 random permutations of each gene set. All genomic and large-scale microarray data have been submitted to the Gene Expression Omnibus (GEO).

### ChIP and high-throughput sequencing

ChIP experiments were performed as previously described.^20^ ChIP-Seq libraries were prepared using the TruSeq DNA Sample Prep Kit v2 (Illumina, San Diego, CA) and sequenced with an Illumina GA2X sequencer. Bowtie2 software^61^ was used to map the sequence reads to the *mm9* mouse genome. Peak calling, motif analysis, and identification of superenhancers were done with HOMER software.^62^ Global annotation of peaks with respect to TSS and gene body was performed with the R Bioconductor package ChIPseeker.^63^ Centered *K*-means clustering was performed using Cluster 3.0 and visualized using Java-Treeview program.^64^ Over layered ChIP-Seq peaks at noted loci were visualized using Genome browser in the Box (GBiB).^65^

### Digital radiography

Images were taken using the LX-60 Laboratory Radiography System (FaxitronBioptics, LLC, Tucson, AZ). Full skeleton images were taken of 4-week-old mice immediately after euthanasia. Skulls and hind limbs were dissected from mice at the ages indicated, soft tissue removed, formalin-fixed for 24 h, and stored in 70% ethanol prior to imaging. Radiographic densities of the femoral diaphysis and distal metaphysis in the mediolateral radiographic view were measured in Hounsfield units (HU) using imaging software (OsiriX, Geneva, Switzerland). HU values were then compared to those of a known BMD calibration phantom (mg·cm^–3^) after correcting for sample thickness (RATOC System Engineering Co, Tokyo, Japan). This allowed for calculation of relative BMDs.

### TRAP staining and bone histomorphometry

Femurs used for radiography were decalcified in 14% EDTA for 2 weeks, embedded in paraffin, and cut to 4 µm thick sagittal sections. Sections were stained for TRAP using a Leukocyte Acid Phosphatase kit (Sigma-Aldrich) to visualize OCs. Stained slides were scanned (Aperio ScanScope XT, Vista, CA) and OC number and surface and trabecular surface measured to calculate Oc.S/BS and Oc.N/BS (Aperio ImageScope software). For TRAP staining of OCs cultured from primary BMDMs or splenocytes, cells were grown on adherent plates in CSF1/ RANKL media for 5 days then fixed with 4% paraformaldehyde for 30 min at 4 °C. Fixed cells were then stained (Sigma-Aldrich). Total cell number and OC number was quantified using FIJI software.^66^ For all TRAP staining, OCs were defined as TRAP positive cells having three or more visible nuclei.

### OCP isolation, culture, and cytometric analysis

OCPs were isolated from either peripheral blood, BM, or spleens of 4–8-week-old mice as indicated. For mediation of myeloid-specific *Pu.1* deletion in mice possessing the *Csf1rTAMCre* allele, *Pu.1*^*fl/fl*^ *Csf1rTAMCre *+ mice and littermate *Pu.1*^*fl/fl*^ controls were injected with 7 daily injections of 1 mg tamoxifen/mouse/day. On day 8, tissues were harvested and processed for OCP sorting. OCPs from the BM were isolated by FACS and cultured for TRAP staining as previously described^29^ and PMNs (CD11b^hi^Ly6C^med^), PIMs (CD11b^med^Ly6C^hi^), MDPs (CD11b^neg^Ly6C^neg^), and OCPs (CD11b^−/lo^Ly6C^hi^) were characterized by flow cytometry using anti-mouse flow cytometry antibodies (CD11b [BD Pharmingen, San Jose, CA] and Ly6C [eBioscience, San Diego, CA]). Owing to the lack of BM space in *Pu.1*^*fl/fl*^ *CtskCre + *mice, spleens were used for OCP isolation. Spleens were mechanically digested, OCPs isolated by FACS as described above, and plated in CSF1/RANKL containing medium. At the indicated time points, cultured OCPs were stained for PU.1 expression by immunocytochemistry as previously described.^67^

To isolate OCPs from circulation, whole-peripheral blood was collected from the subclavian artery in PBS containing 50 mmol·L^–1^ EDTA. After red blood cell (RBC) lysis with ammonium chloride, cells were processed with the Mouse Monocyte Enrichment Kit (StemCell Technologies, Vancouver, Canada). Cells were labeled and sorted identical to the BM preparations; however, an alternative CD11b^-/lo^Ly6C^hi^CD115^+^ OCP signature was used (eBioscience). Flow sorting or FACS were performed on either a LSRII flow cytometer or a BD FACS Aria and analysis performed using FlowJo software (FlowJo LLC, Ashland, OR).

### Chromosome conformation capture (3C)

The 3C assay was performed as previously described.^68^ *Bgl*II was used for restriction enzyme digestion. Bacterial artificial chromosome RP24-215E4 (Children’s Hospital of Oakland Research Institute BacPac Resources, Oakland, CA) encompassing -27.5 kilobases to +147 kilobases from the TSS of *Nfatc1* was used as a positive control to prepare standards for chimeric products. For all 3C experiments, 1 × 10^7^ BMDMs cells were used. Only samples that had a restriction digestion efficiency of 70% and above were further processed. Primer and probe sequences are listed (Supplemental Table 6).

### Statistical analysis

All data points reported represent biological replicates and all in vitro and in vivo experiments were performed a minimum of three times to ensure reproducibility of results. A sample size of *n* = 3–4 was chosen for all comparisons with no prior sample size calculations. No animals or biological replicates were excluded from any analysis. Mice were randomly chosen after genotyping for in vitro and in vivo experiments and for all analyses using manual measurements (imaging and histomorphometry), the evaluator was blinded to sample identification.

Data are expressed as mean ± 1 standard deviation. Data distribution and variance were initially assessed. Non-normally distributed data and/or data with unequal variance underwent log10 transformation of which all data were then normally distributed and had equal variance. Therefore, parametric statistical comparisons were used for all analyses in the manuscript—comparisons between two groups were performed with an unpaired *t*-test and between three or more groups were made with a one-way ANOVA and Newman–Keuls post-hoc analysis. Statistical comparisons for gene expression and ChIP analyses were conducted using the ∆Ct values. All analyses were performed with Prism 7 (GraphPad Software, Inc., La Jolla, CA) and SigmaStat v3.5 (Systat Software, Inc.) with statistical significance established at *P* < 0.05. For all *t*-tests and ANOVAs, *t* and d*F* or *F* and d*F* values, respectively, are reported in Supplementary Table 7.

## Electronic supplementary material

Supplemental Figures and Tables(DOCX 3252 kb)

Supplemental_Tables.xlsx(XLSX 8434 kb)
