# Supplementary material for: Enhancer variants reveal a conserved transcription factor network governed by PU.1 during osteoclast differentiation
Source: Bone Res. 2018 Mar 28;6:8. doi: 10.1038/s41413-018-0011-1 (PMC5874256; doi:10.1038/s41413-018-0011-1)
Supplement: Supplementary file 1 — Supplemental Figures and Tables(DOCX 3252 kb) [file 41413_2018_11_MOESM1_ESM.docx]

**Supplemental Methods/References, Figures, and Tables**

**Supplementary Methods. Bioinformatics analysis.**

1. **General processing of ChIP-Seq data from external resources.** All external ChIP-seq datasets were either downloaded from GEO omnibus or European nucleotide archive (See URLS section) as fastQ file format. We used Bowtie2^1^ software to align the external data to mm9 or hg19 version of the mouse and human genomes respectively. Peak calling, motif analysis and annotation to the nearest promoter or TSS was performed using Homer software suite^2^. Global annotation of peaks with respect to TSS and gene body was performed with R Bioconductor package, ChIPseeker^3^. For visualization of tag density plots, the tag density around selected regions were created using Homer suite, the centered K-means clustering was performed using cluster 3.0 package^4^ and visualized using Java-Treeview^5^ program. Visualization of multiple ChIP-Seq peaks at noted loci as a hub track was achieved by converting the tag density data in to a multiwig files using Homer suite and visualizing in Genome browser in the Box (GBiB)^6^.
2. **Identification of BMD-SNPs and associated top enriched processes**. The meta-analysis^7^ from GEFOS consortium with a p value cutoff of 0.05 was converted to hg19 coordinates using VCF-tools^8^. PU.1 as well as RUNX2 ChIP-Seq data with corresponding H3K27Ac data from the external resources were intersected using Bedtools^9^ software to identify PU.1/RUNX2 bound enhancers. Similarly, the BMD-SNPs intersecting PU.1 or RUNX2 bound enhancers were isolated. The gene symbols segregated by above filtering methods were utilized to evaluate the enriched processes using Metascape^10^ and or “Toppgene” web algorithms. The top networks were visualized and organized by Cytoscape 3 (see URLs section) software.
3. **Orthologous comparison of temporal expression during human and mouse OC differentiation**. Orthologous comparison of expression kinetics during human and mouse osteoclast differentiation was performed using Affymetrix HGU133 and Mouse 430 Gene Chips, respectively (Affymetrix, Santa Clara, CA). Briefly, the gene symbols were collapsed using the probeset value with highest mean signal. The all gene symbols that represent transcription cluster were collapsed in to unique identifiers and their respective expression values were retained. In both human and mouse expression data only the genes that follow similar expression kinetics were retained and clustered over eight samples using centered K means clustering.

**Supplementary References**

1. Langmead, B. & Salzberg, S.L. Fast gapped-read alignment with Bowtie 2. *Nat Methods* **9**, 357-359 (2012).

2. Heinz, S.*, et al.* Simple combinations of lineage-determining transcription factors prime cis-regulatory elements required for macrophage and B cell identities. *Mol Cell* **38**, 576-589 (2010).

3. Yu, G., Wang, L.G. & He, Q.Y. ChIPseeker: an R/Bioconductor package for ChIP peak annotation, comparison and visualization. *Bioinformatics* **31**, 2382-2383 (2015).

4. de Hoon, M.J., Imoto, S., Nolan, J. & Miyano, S. Open source clustering software. *Bioinformatics* **20**, 1453-1454 (2004).

5. Saldanha, A.J. Java Treeview--extensible visualization of microarray data. *Bioinformatics* **20**, 3246-3248 (2004).

6. Haeussler, M.*, et al.* Navigating protected genomics data with UCSC Genome Browser in a Box. *Bioinformatics* **31**, 764-766 (2015).

7. Estrada, K.*, et al.* Genome-wide meta-analysis identifies 56 bone mineral density loci and reveals 14 loci associated with risk of fracture. *Nat Genet* **44**, 491-501 (2012).

8. Danecek, P.*, et al.* The variant call format and VCFtools. *Bioinformatics* **27**, 2156-2158 (2011).

9. Quinlan, A.R. BEDTools: The Swiss-Army Tool for Genome Feature Analysis. *Curr Protoc Bioinformatics* **47**, 11 12 11-34 (2014).

10. Tripathi, S.*, et al.* Meta- and Orthogonal Integration of Influenza "OMICs" Data Defines a Role for UBR4 in Virus Budding. *Cell Host Microbe* **18**, 723-735 (2015).


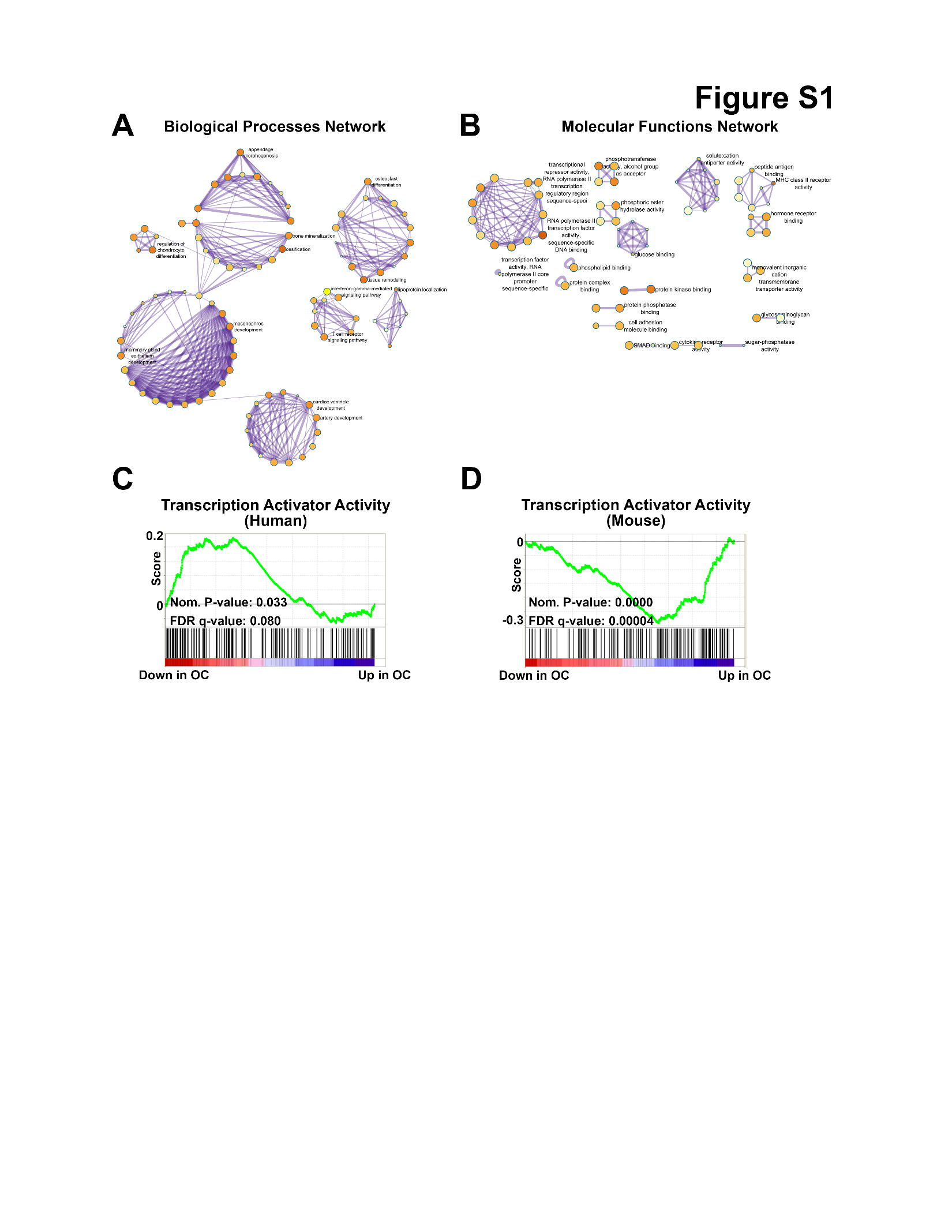
**Supplemental Figure 1:** (A) Network of biological processes enriched in PU.1 BMD-SNP genes. (B) Network of molecular functions enriched in PU.1 BMD-SNP genes. (C) GSEA analysis of molecular function from human OC differentiation kinetics. (D) GSEA analysis of molecular function from mouse OC differentiation kinetics.





**Supplemental Figure 2:** (A) Genotyping PCR to amplify the *Pu.1^KO^* allele in DNA extracted from *in vitro* differentiated OCs from *Pu.1^fl/fl^*;*CtskCre+* and *Pu.1^fl/fl^* control spleens. (B) Representative immunofluorescence images and quantification of PU.1 nuclear fluorescence during the course of OC differentiation from CD11b^-/lo^Ly6C^hi^ OCPs from *Pu.1^fl/fl^*;*CtskCre+* spleens. Scale bars = 20 μm. (C) Representative dorsoventral whole body images of 4 week old male littermate *Pu.1^fl/fl^*;*CtskCre+* (*Pu.1^ΔOC/ΔOC^*) mice and *Pu.1^fl/fl^* controls. Image is representative of n=3 pairs. (D) Representative ventrodorsal whole skeleton digital radiographs of 8 day old littermate *Pu.1^ΔOC/ΔOC^* mice and *Pu.1^fl/fl^* controls. Image is representative of n=3 pairs. Scale bar = 10 mm. (E) Representative ventrodorsal images of the heads of 4 week old male littermate *Pu.1^ΔOC/ΔOC^* mice and *Pu.1^fl/fl^* controls. Image is representative of n=3 pairs. (F) Representative images and quantification of TRAP positive multinuclear cells (MNCs; > 3 nuclei/cell) of *in vitro* differentiated OCs from *Pu.1^ΔOC/ΔOC^* and *Pu.1^fl/fl^* spleens (n=3)*.* (G) Timeline of tamoxifen induction of myeloid-specific *Pu.1* deletion and blood and bone marrow (BM) harvest for flow cytometry and FACS. (H) Western blot of nuclear extracts from BMMs confirming *Pu.1* deletion (*Pu.1 ^ΔMP/ΔMP^*) upon *Csf1rTAMCre* activation (n=3). Nuclear LAMIN B is the loading control. (I) Genotyping PCR to amplify the *Pu.1^fl^*, *Pu.1^WT^* , and *Pu.1^KO^* alleles in DNA extracted from tail biopsies, BM, and BMMs from *Pu.1^fl/fl^;Csf1rTAMCre+* mice and *Pu.1^fl/+^* controls. Mice were given seven daily intraperitoneal injections of 1 mg tamoxifen before harvest. BMMs were also treated *in vitro* with 4-OHT for three days prior to harvest. Water only control PCR is labelled ‘W’. (J) Quantification of the CD11b^-/lo^Ly6C^hi^CD115^+^ OCP population in the blood of 6-8 week old *Pu.1^ΔMP/ΔMP^* mice and *Pu.1^fl/fl^* controls (n=3). (K) Quantification of common monocyte dendritic cell precursors (MDPs), granulocytes (PMNs), and pro-inflammatory monocytes (PIMs) from the BM of 6-8 week old *Pu.1^ΔMP/ΔMP^* mice and *Pu.1^fl/fl^* controls (n=4).


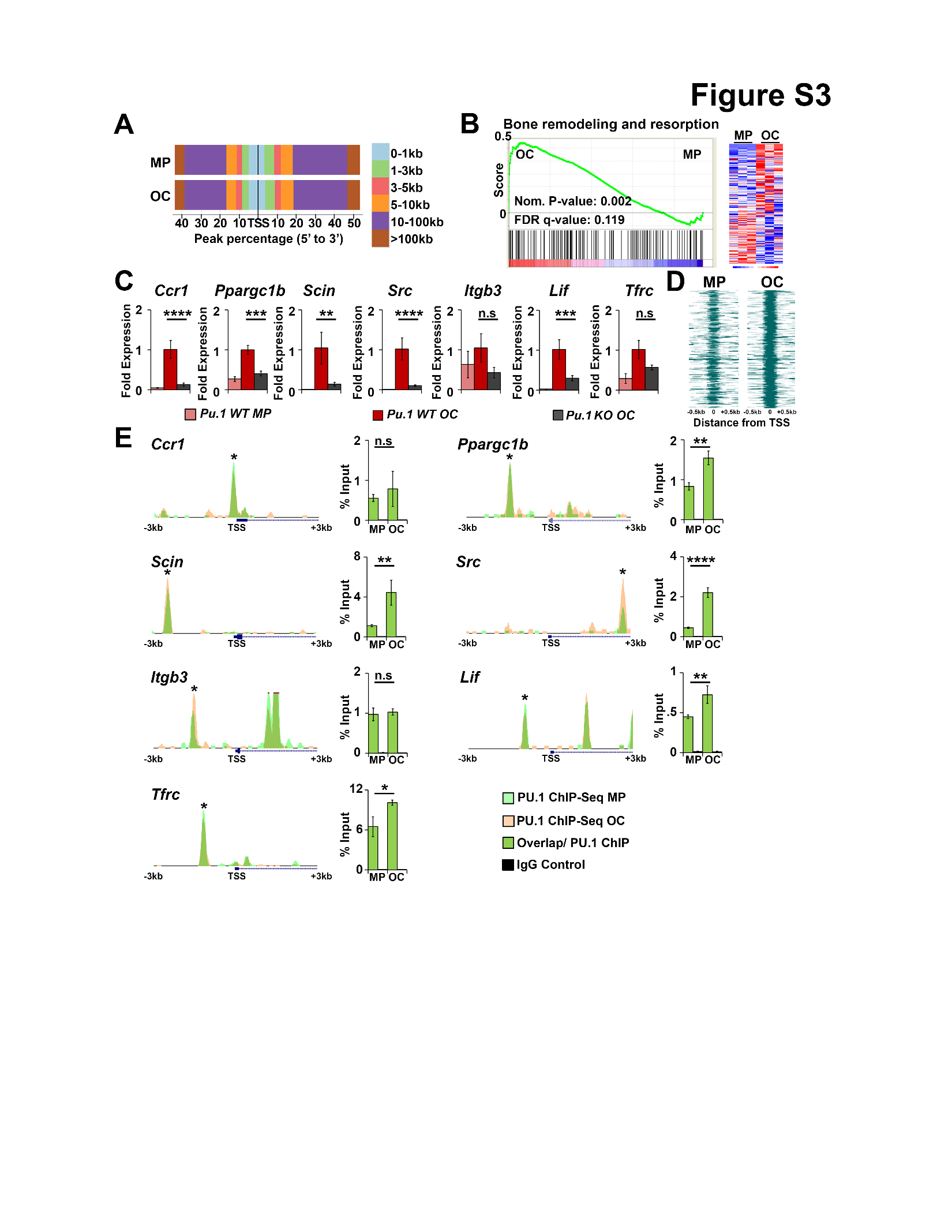
**Supplemental Figure 3:** (A) Distribution of PU.1 peak distances from known TSSs. (B) GSEA plot of a bone remodeling and resorption gene set significantly enriched in genes with PU.1 OC peaks using our MP and OC microarray data (n=3). Heatmap (right) indicating MP and OC expression of genes in the gene list. (C) RT-qPCR analysis of genes on the bone remodeling and resorption GSEA gene list involved in OC function. Gene expression is shown for WT MPs and OCs and *Pu.1 KO* OCs (n=3). (D) Treeview plot of PU.1 MP and OC tags ± 500 base pairs from the TSSs of all genes on the GSEA list. (E) Depiction of MP and OC PU.1 ChIP-Seq peaks ± 3 kb from the TSS of 7 genes on the GSEA list*.* Conventional ChIP validation of PU.1 binding to the starred sites is shown (bar graphs, n=3).


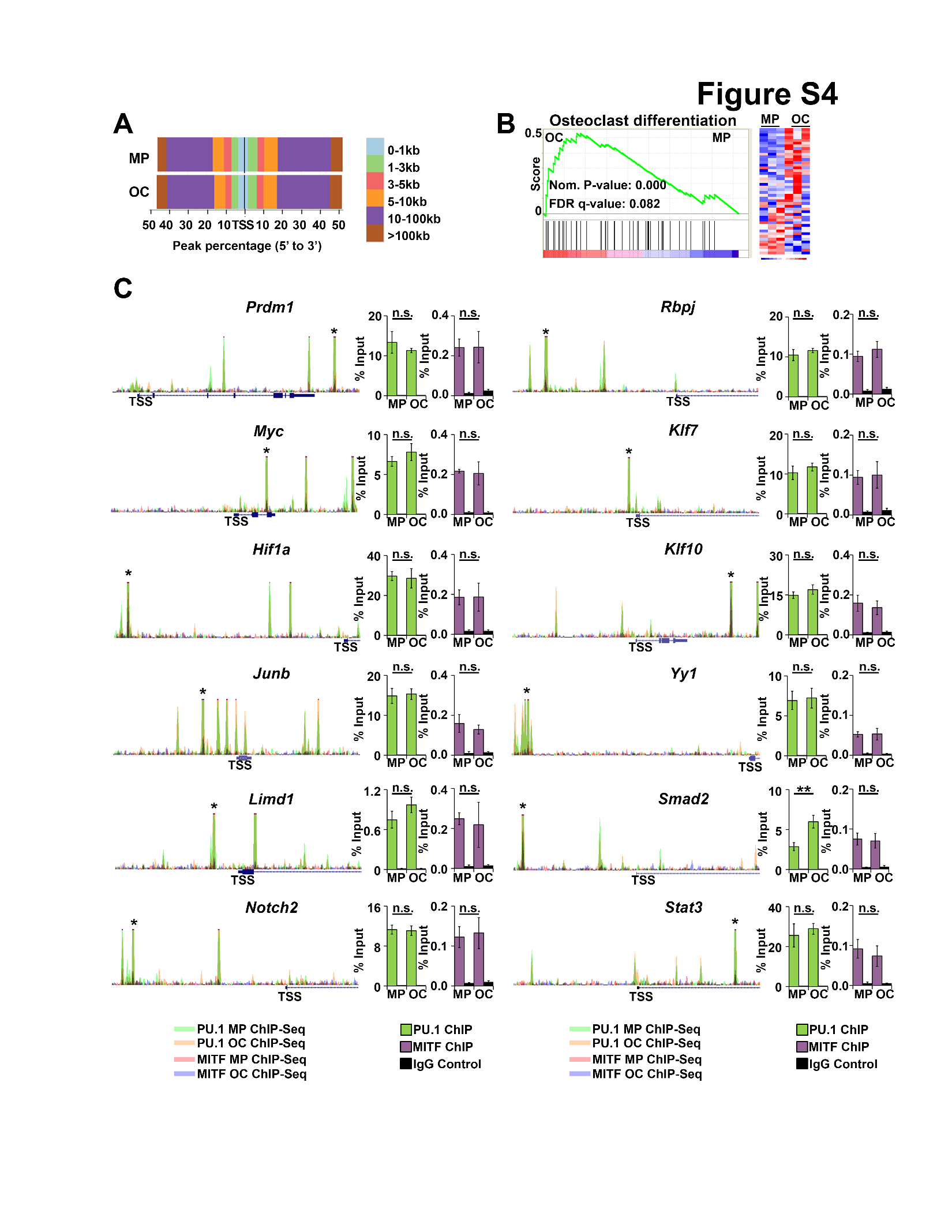
**Supplemental Figure 4:** (A) Distribution of MITF peak distances from known TSSs. (B) GSEA plot of an OC differentiation gene set significantly enriched in genes with PU.1/MITF overlapping OC peaks using our MP and OC microarray data (n=3). Heatmap (right) indicating MP and OC expression of genes in the gene list. (C) Depiction of MP and OC PU.1 and MITF ChIP-Seq peaks at 12 more TF loci with roles in OC differentiation from the GSEA list. Each trace is 30 kb wide and the TSS is located as indicated. Conventional ChIP validation of PU.1 and MITF binding to the starred sites is shown (bar graphs, n=3).


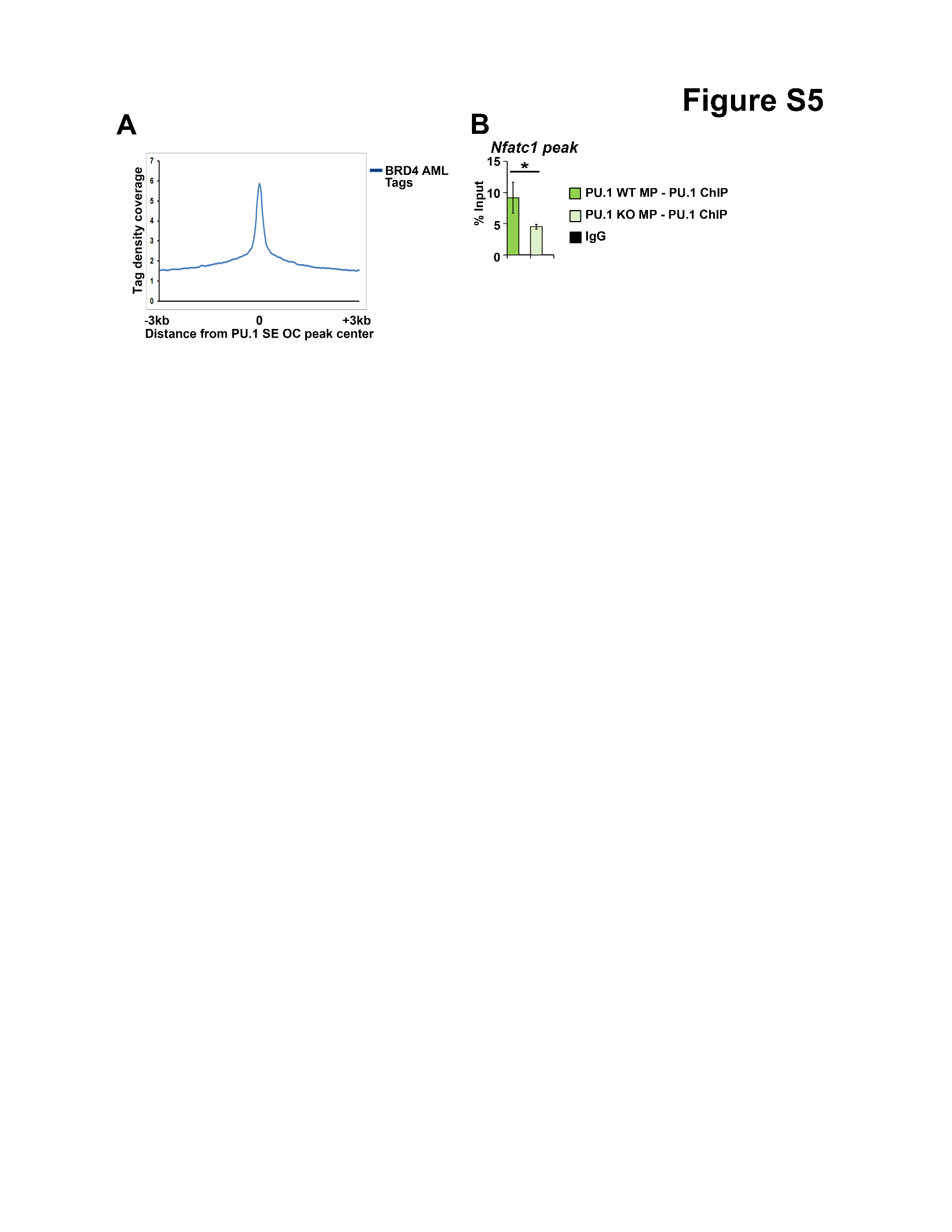
**Supplemental Figure 5:** (A) ChIP-Seq tag density coverage of murine AML BRD4 (blue) ± 3 kb from PU.1 superenhancer OC peak centers. (B) Conventional ChIP of PU.1 binding to the *Nfatc1* peak starred in Figure 4A in WT and *Pu.1 KO* MPs (n=3).

**Supplemental Table 1:** Unique RUNX2-Enh-BMD-SNPs and PU.1-Enh-BMD-SNPs from the six groups in the GEFOS consortium (See attached Excel file).

**Supplemental Table 2:** Annotated MITF and PU.1 ChIP-Seq peaks from murine myeloid precursors and osteoclasts (See attached Excel file).

**Supplemental Table 3:** Microarray data and GSEA analysis results from this study (See attached Excel file).

**Supplemental Table 4:** Biological processes enriched in PU.1-occupied loci by GSEA (This document, below).

| **RANK** | **Biological Process** | **ES^a^** | **NES^b^** | **NOM p-val^c^** | **FDR q-val^d^** |
| --- | --- | --- | --- | --- | --- |
| 1 | BONE_RESORPTION | 0.79 | 2.18 | 0 | 0.001 |
| 2 | OSTEOCLAST_DIFFERENTIATION | 0.62 | 2.07 | 0 | 0.004 |
| 3 | MYELOID_CELL_DIFFERENTIATION | 0.72 | 1.85 | 0.004 | 0.15 |
| 4 | BONE_REMODELING_&_RESORPTION | 0.54 | 1.85 | 0.002 | 0.119 |
| 5 | TOLL-LIKE_RECEPTOR_4_SIGNALING_PATHWAY | 0.57 | 1.83 | 0.002 | 0.127 |

**^a^**Enrichment Score, **^b^**Normalized Enrichment Score, **^c^**Nominal p-value, **^d^**False Discovery Rate q-value

**Supplemental Table 5:** Superenhancers marked by PU.1 in OCs (See attached Excel file).

**Supplemental Table 6:** List of oligonucleotides used in the study (This document, below).

| **Primers used for ChIP-seq Validation by q-PCR** | | | |
| --- | --- | --- | --- |
| **Gene** | **Forward Primer** | **Reverse Primer** | **UPL Probe #** |
| *Acp5* | Tgggctaaatagggaggactg | cccctgggattatgttctttt | *64* |
| *Bcl6* | Gagggagggacttgagtatttg | tcaagtttttccactcctagcc | *108* |
| *Ccr1* | Aggtagctctgccctacaagaa | tcattgtcacacacagagttcaa | *58* |
| *cFos* | Gagtctgaacttagccttgtctca | gtgacttgggaaagccttttt | *60* |
| *Ctsk* | Cagactcttatgatctgtcacatgc | cccaaactaggtccagttacaag | *60* |
| *Fosl2* | Tgcccacttaaccacagaca | aggaacacagcccagacact | *6* |
| *Hif1a* | Tgaaagaggaagtgggctga | aatgaaagggaaggcactagc | *84* |
| *Irf8* | Tcgctgtctgcttcagagg | gcaaacactaactatcggcaaga | *29* |
| *Itgb3* | Ctctcactaagcctggagctg | ccagtgcaatgggaggaa | *29* |
| *Junb* | Ccaaggtgtcccctttaaga | catcacccctacttccctga | *6* |
| *Klf7* | Aagtgccagagaggaagtgc | agctagtctccgtgcagctc | *53* |
| *Klf10* | Ggcctcttcaagtcactggt | acgaagggagggagtgga | *12* |
| *Lif* | Ggactaatattgctctaggcaggt | agggagggaagggaaagg | *91* |
| *Limd1* | Gaggaaatgcctacgattgg | gagcactggctctaatcagga | *68* |
| *Myc* | Ggcgtggctaactgtgatct | ccacatcaatttcttcctcatct | *69* |
| *Nfact1* | Ggtgtctgaagcccagagaa | ccacatggtcttttccacaac | *6* |
| *Notch2* | Ggacttttgcaaccacttcc | gcagtaaggctactagattttcaattt | *60* |
| *Oscar* | Ccacgagtacctatttgatttatcatt | gggcaacaagtacttttatctgc | *67* |
| *Ppargc1b* | Gcaggctccttccagagag | aggaagctgttgcctagtgg | *38* |
| *Prdm1* | Tgcttatctgccacttcctct | acaagtgtgacatagtcatctttgagt | *6* |
| *Rbpj* | Tttggactttgggaagtgga | ccgctattcatttttctcagttct | *60* |
| *Runx1* | Ctgacttctcatctgcctagctt | tcccctttcagggggtatta | *60* |
| *Scin* | Aagtcaatgtaagcttcaatactggat | ccaagctgtcagtggaatca | *69* |
| *Smad2* | Gccttcctctctgacacttcc | cttgatgaacttccagctcaact | *69* |
| *Src* | Ggaagaagctaggtttagtaggagttc | ctgatccttgggctctcagt | *106* |
| *Stat3* | Tctctcaaccagagggaacc | ccctctctataaaatgggaaagact | *40* |
| *Tfrc* | Gtctatagccggaatcattatttctt | ggaagaagagggggtaagga | *63* |
| *Yy1* | Tgcaagcagcatttcctgta | tgcatgccaacgtcacat | *69* |
| **Primers used for mRNA quantitation by q-PCR** | | | |
| **Gene** | **Forward Primer** | **Reverse Primer** | **UPL Probe #** |
| *Acp5* | Cgtctctgcacagattgcat | aagcgcaaacggtagtaagg | 60 |
| *Bcl6* | Ctgcagatggagcatgttgt | gccatttctgcttcactgg | 4 |
| *Ccr1* | Tggacaaaatactctggaaacaca | tgtgaaatctgaaatctccatcc | 73 |
| *cFos* | Cagcctttcctactaccattcc | acagatctgcgcaaaagtcc | 67 |
| *Ctsk* | Cgaaaagagcctagcgaaca | tgggtagcagcagaaacttg | 18 |
| *Fosl2* | Gccgcagaaggagagatg | tttctctccctccggattc | 63 |
| *Irf8* | Gagccagatcctccctgact | ggcatatccggtcaccagt | 26 |
| *Itgb3* | Tgaccggaaggaatttgcta | acagcgggttgtttgctg | 21 |
| *Lif* | Tgaaaacggcctgcatcta | agcagcagtaagggcacaat | 25 |
| *Nfatc1* | Tccaaagtcattttcgtgga | ctttgcttccatctcccaga | 50 |
| *Oscar* | Tctgccccctatgtgctatc | tagtccaaggagccagaacc | 18 |
| *Ppargc1b* | Ctccagttccggctcctc | ccctctgctctcacgtctg | 17 |
| *Rpl4* | Gatgagctgtatggcacttgg | cttgtgcatgggcaggtta | 38 |
| *Runx1* | Ctccgtgctacccactcact | atgacggtgaccagagtgc | 77 |
| *Scin* | Ccagcacaacatggtgga | cactgttctctacacgccagat | 21 |
| *Src* | Tgagccaggatctgaacca | tcctgctccgtgtcccta | 32 |
| *Tfrc* | Tcctttccttgcatattctgg | cccaaataaggatagtctgcatc | 3 |
| **Primers used in 3C assay** | | | |
| **Fragment** | **Primer Sequence** |  |  |
| +T1 | TAG ATT GGG AAG TGC TAG GC |  |  |
| +T2 | CAC AGC CAT TCC TTC CTT TA |  |  |
| +T3 | TAG TTT GGG ACT GGG AAG AA |  |  |
| +T4 | ACA GGG CAG ATA CCT TCT A |  |  |
| +T5 | AAT GTG TTT AGA ACC GCT GAA |  |  |
| -T1 | AGA ACC CTG CTT TTG CAC TA |  |  |
| -T2 | GCT CAC ATA CAG AGG AGC TG |  |  |
| -T3 | AGG GGT CTT CAC CAC AAC T |  |  |
| -T4 | TTC ATT TCC CAG CTC CTT C |  |  |
| -T5 | CCA CAG TAA ATG GAT TGC TTC |  |  |
|  |  |  |  |
| **Constant Primer** | CAT ATG TCC GCC CAT AAG AC |  |  |
| **Probe** | TC TTC CAG TC TTG CCT GCC CTC AT |  |  |

**Supplemental Table 7:** Statistical justification (See attached Excel file).

**Supplemental Table 8:** External datasets and accession numbers used in this study (This document, below).

| Sample | Accession | Genome |
| --- | --- | --- |
| Human PBMC Control IgG | GSM785497 | hg19 |
| Human PBMC PU.1 | GSM785495 | hg19 |
| Human PBMC H3K27Ac | GSM785494 | hg19 |
| Human macrophage PU.1 | GSM785501 | hg19 |
| Human monocyte H3K27Ac | GSM785494 | hg19 |
| Human macrophage H3K27Ac | GSM785500 | hg19 |
| Human iMSC RUNX2 | ERR329086 | hg19 |
| Human iMSC H3K27Ac | ERR329095 | hg19 |
| Mouse BMDM H3K4me3 | GSM1000065 | mm9 |
| Mouse BMDM H3K4me1 | GSM1000066 | mm9 |
| Mouse BMDM Input | GSM1000206 | mm9 |
| Mouse BMDM H3K27Ac | GSM1000074 | mm9 |
| Mouse MITF Mast cells | GSM1167584 | mm9 |
| Mouse MLL PU.1 | GSM1614785 | mm9 |
| Mouse MLL BRD4 | GSM1614770 | mm9 |
